# Universal redirection of CAR T cells against solid tumours via membrane-inserted ligands for the CAR

**DOI:** 10.1038/s41551-023-01048-8

**Published:** 2023-06-08

**Authors:** Angela Q. Zhang, Alexander Hostetler, Laura E. Chen, Vainavi Mukkamala, Wuhbet Abraham, Lucia T. Padilla, Alexandra N. Wolff, Laura Maiorino, Coralie M. Backlund, Aereas Aung, Mariane Melo, Na Li, Shengwei Wu, Darrell J. Irvine

**Affiliations:** 1 Koch Institute for Integrative Cancer Research, Cambridge, MA USA; 2 Department of Health Sciences and Technology, Massachusetts Institute of Technology, Cambridge, MA USA; 3 Department of Biophysics, Harvard University, Cambridge, MA USA; 4 Department of Biological Engineering, Massachusetts Institute of Technology, Cambridge, MA USA; 5 Ragon Institute of MIT, MGH, and Harvard, Cambridge, MA USA; 6 Howard Hughes Medical Institute, Chevy Chase, MD USA

**Keywords:** Cancer immunotherapy, Cancer immunotherapy, Drug delivery

## Abstract

The effectiveness of chimaeric antigen receptor (CAR) T cell therapies for solid tumours is hindered by difficulties in the selection of an effective target antigen, owing to the heterogeneous expression of tumour antigens and to target antigen expression in healthy tissues. Here we show that T cells with a CAR specific for fluorescein isothiocyanate (FITC) can be directed against solid tumours via the intratumoural administration of a FITC-conjugated lipid–poly(ethylene)-glycol amphiphile that inserts itself into cell membranes. In syngeneic and human tumour xenografts in mice, ‘amphiphile tagging’ of tumour cells drove tumour regression via the proliferation and accumulation of FITC-specific CAR T cells in the tumours. In syngeneic tumours, the therapy induced the infiltration of host T cells, elicited endogenous tumour-specific T cell priming and led to activity against distal untreated tumours and to protection against tumour rechallenge. Membrane-inserting ligands for specific CARs may facilitate the development of adoptive cell therapies that work independently of antigen expression and of tissue of origin.

## Abstract (web summary)

T cells with a chimaeric antigen receptor specific for fluorescein isothiocyanate can be directed against solid tumours via the intratumoural administration of a fluorescein isothiocyanate-conjugated amphiphile that inserts itself in cell membranes.

## Main

Chimaeric antigen receptor (CAR) T cell therapy has achieved clinical success in the treatment of multiple haematological malignancies^1–3^, including most recently multiple myeloma^4^; yet its translation to solid tumours has been challenging^5–7^. One of the main challenges presented by solid tumours is target antigen selection. Current CARs target overexpressed self-antigens or mutated cell surface proteins expressed by cancer cells; however, antigen selection for targeting solid tumours is often problematic. On the one hand, tumour-associated antigens, such as human epidermal growth factor receptor 2 (HER2) or mesothelin, are often also expressed in healthy tissues, leading to on-target off-tumour toxicities^8–10^. On the other hand, the expression of mutated cell surface proteins, such as epidermal growth factor receptor III (EGFRvIII), is restricted to cancer cells, but such neoantigens are often heterogeneously expressed, increasing the likelihood of resistance and/or outgrowth of antigen-negative cancer cells^11^. Thus, there is a great need to identify more optimal tumour antigens or to develop alternative strategies to direct CAR T cells against solid tumours. Examples of recent advances include the optimization of CAR designs to enhance the recognition of antigen-low tumour cells^12,13^, multi-specific CARs that recognize more than one antigen^13–15^, and genetic circuits enabling antigen-sensing logic (such as synNotch circuits to limit CAR expression to the tumour^16–19^). However, these combinatorial approaches intrinsically become more complex with each additional antigen, and understanding how such CAR T cell genetic circuits should be tuned to be safe and effective in the face of varied antigen levels across patients remains a complex translational problem.

One immunological mechanism by which CAR T cell therapy can combat tumour antigenic heterogeneity and antigen loss is via the phenomenon of antigen spreading (or epitope spreading), whereby CAR T cell killing of cancer cells promotes the release of tumour antigens in a favourable pro-inflammatory microenvironment, leading to priming of de novo endogenous T cell responses targeting additional antigens in the tumour^20^. Evidence of epitope spreading has been observed in murine preclinical studies including EGFRvIII CAR T cells in murine glioma^21,22^, interleukin 13 receptor subunit alpha 2 (IL-13Ra2) CAR T cells in murine glioblastoma multiforme^23^, and CD19 CAR T cells targeting solid tumours induced to express CD19 (ref. ^24^). To enhance antigen spreading, T cell receptor-transgenic and CAR T cells have been engineered to secrete FMS-like tyrosine kinase 3 ligand (Flt3L) to expand cross-presenting dendritic cells (DCs), which are critical to this process^25^.

Another approach to the problem of antigen selection in solid tumours has been to therapeutically induce exogenous antigens on tumour cells to enable CAR T cell targeting. For example, viral gene-therapy vectors or oncolytic viruses have been used to transduce tumour cells with arbitrary foreign antigens or antigens with a known side-effect profile in CAR T cell therapy (such as CD19) (refs. ^24,26,27^). This is an attractive approach because antigens can be selected with no concern of cross-targeting of healthy tissue, and the induction of antigen spreading by CAR T cell attack on transduced tumours promotes endogenous T cell responses that limit antigen-loss escape and provide systemic antitumour immunity^24,27^. However, a translational limitation of introducing foreign antigens via engineered viruses is the heterogeneity and highly variable transduction achieved by viral vectors both within individual tumours and across tumours from patient to patient^28–30^.

We have extensively studied the behaviour of amphiphilic molecules composed of therapeutic compounds conjugated to amphiphilic poly(ethylene glycol) (PEG)-lipids. These ‘amph-ligand’ conjugates exhibit several useful properties for controlling the pharmacokinetics and biodistribution of linked therapeutic payloads such as peptides, proteins or small molecules^31–33^. For example, amph-ligands can insert their lipid tails into the plasma membrane of cells in vitro and in vivo without toxicity, leading to the cell surface display of the attached compounds^21,31^. Following parenteral injection, amph-ligands also efficiently traffic into draining lymph nodes (LNs) and decorate the surfaces of antigen-presenting cells (APCs)^21^. We previously exploited this physical chemistry to decorate LN APCs with ligands for CAR T cells, creating a vaccinal effect in draining LNs that enhanced the proliferation, function and efficacy of CAR T cells^21^.

In this Article, we report a strategy to employ amph-ligands as a means to redirect CAR T cells against any solid tumour. We show that an amphiphile conjugate of the small molecule fluorescein isothiocyanate (FITC) added to cancer cells in vitro leads to plasma membrane decoration of the tumour cells, enabling recognition by CAR T cells bearing FITC-specific chimaeric receptors and triggering CAR T cell activation, proliferation and killing of the amph-FITC-decorated target cells. In vivo, intratumoural (i.t.) injection of amph-FITC followed by systemic infusion of FITC-specific CAR T cells leads to CAR T cell activation and accumulation in tumours, accompanied by tumour regression in models of murine and human xenograft solid tumours. Amph-FITC-stimulated CAR T cell activity also triggered priming of an endogenous tumour-specific T cell response, inducing ‘abscopal’ responses against distal untreated tumours and immune memory against tumour rechallenge. Hence, amph-FITC-directed CAR T cells may provide an approach for safe and effective tumour regression and for systemic antitumour immunity that can be applied to any solid tumour, irrespective of tissue of origin or malignant genotype.

## Results

### Amph-FITC tags tumour cells for destruction by CAR T cells

**Fig. 1:**
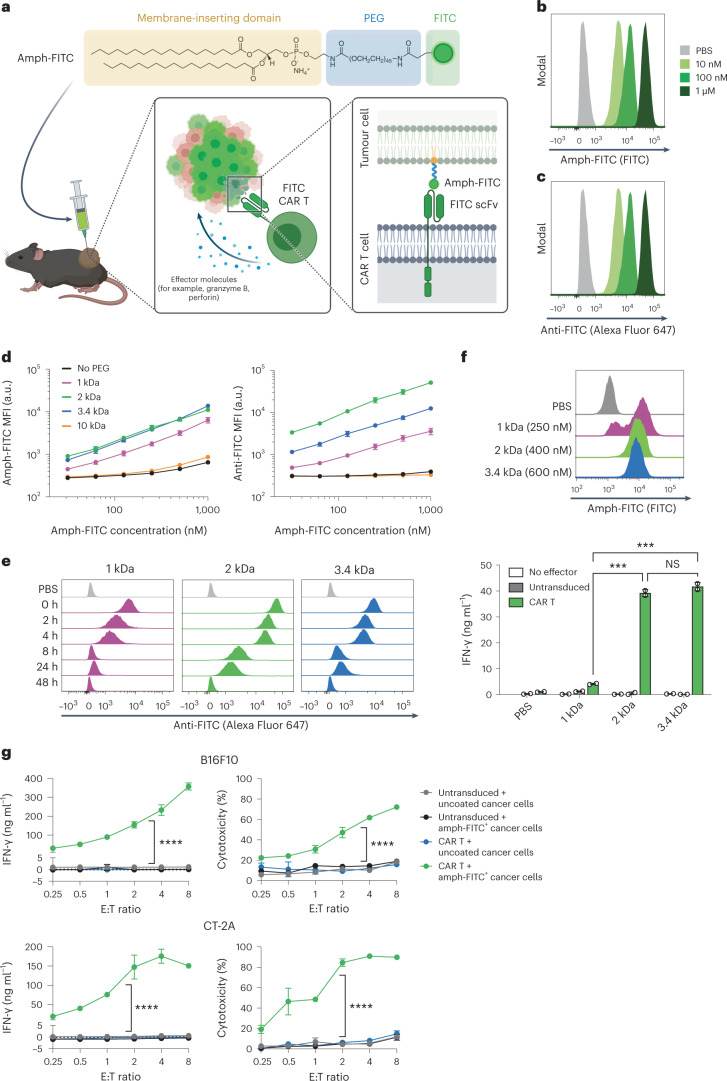
Amph-FITC tags cancer cells for CAR T cell-mediated killing. **a**, Schematic of amph-FITC structure, insertion into cancer cell membranes, and recognition by FITC CAR T cells. Created with Biorender.com. **b**,**c**, B16F10 murine melanoma cells were incubated with amph-FITC at indicated concentrations for 30 min at 37 °C in PBS, stained with an anti-FITC antibody and analysed by flow cytometry. Shown are representative histograms of total FITC (**b**) and anti-FITC signals (**c**). **d**, B16F10 cells were incubated with amph-FITC conjugates of varying PEG MW as in **b** at the indicated concentrations and then stained with anti-FITC for flow cytometry analysis. Shown are median fluorescence intensities (MFIs) of FITC (left) and anti-FITC (right) signals. **e**, Kinetic analysis of anti-FITC MFI of B16F10 cultured in RPMI following tagging in 100 nM amph-FITC. **f**, Histograms of MC38 colon cancer cells labelled with titrated concentrations of amph-FITC to yield the same FITC fluorescence per cell (top). Cancer cells were subsequently cultured with 4m5.3-28z CAR T cells at a 1:1 E:T ratio. **g**,**h**, Co-culture of FITC-specific E2-m28z CAR T cells or control untransduced T cells with B16F10 (**g**) or CT-2A (**h**) cancer cells with or without coating by 100 nM amph-FITC at a range of E:T ratios. Shown are mean ± s.d. from duplicate samples (all biological replicates). *P* values were determined by two-way ANOVA. NS, not significant; ***P < 0.001, ****P < 0.0001. Source data

We envisioned that the membrane-inserting properties of PEG-lipid conjugates could be used to decorate tumour cells with ligands for a CAR, ‘painting’ the tumour to be recognized by CAR T cells. In this setting, the choice of CAR T cell ligand is arbitrary, and we chose to use FITC as a safe, non-immunogenic compound to redirect CAR T cells against tumours. We hypothesized that i.t. administration of 1,2-distearoyl-*sn*-glycero-3-phosphoethanolamine-PEG-FITC (DSPE-PEG-FITC, or amph-FITC) would enable recognition and killing of tumour cells by FITC-specific CAR T cells (Fig. 1a).

We first evaluated labelling of cancer cells in vitro by incubating amph-FITC with B16F10 murine melanoma cells for 30 min at 37 °C. Flow cytometry analysis of the melanoma cells revealed dose-dependent association of amph-FITC with the cells (Fig. 1b), and FITC was exposed on the cell surfaces as revealed by subsequent staining of amph-FITC-coated cells with an anti-FITC antibody (Fig. 1c). Similarly, amph-FITC could also associate with and be exposed on the surfaces of MC38 murine colon carcinoma cells and CT-2A murine glioblastoma cells (Supplementary Fig. 1a,b). It is known that the physical location of CAR epitopes on target antigens (closer or farther from the cell membrane) impacts chimaeric receptor signalling and CAR T activation^34,35^. In addition, we have previously shown that the length of the polymer chain on PEG-DSPE amphiphiles impacts membrane insertion^32^. To determine how the PEG linker length of amph-FITC affects membrane insertion and stability of labelling, we incubated B16F10 cells with amph-FITC molecules composed of a range of PEG molecular weights (MW) and measured the resultant total cell-associated and cell surface-exposed FITC at a range of concentrations (Fig. 1d), then washed the cells into fresh medium and tracked loss of FITC signal over 48 h (Fig. 1e and Supplementary Fig. 1c). DSPE-PEG_2k_-FITC achieved the highest insertion at low concentrations and exhibited the greatest persistence, with substantial labelling still detectable after 24 h in fresh medium.

We next tested CAR T cell recognition of B16 cells decorated with amph-FITC molecules. FITC-specific CAR T cells were generated by transducing primary murine CD8^+^ T cells with a CAR composed of the FITC-specific single-chain variable fragments (scFvs) 4m5.3 (ref. ^36^) or E2 (ref. ^37^) fused to a CD8 hinge, CD8 transmembrane domain, CD28 co-stimulatory domain and CD3ζ signalling domain similar to our previously reported designs^21^ (abbreviated hereafter as the 4m5.3-28z and E2-28z CARs, Supplementary Fig. 2a–d). To assess how PEG linker length influences that ability of FITC-specific CAR T cells to recognize amph-FITC-decorated target cells, we incubated MC38 colon cancer cells with amph-FITC of varying MW at concentrations titrated to give the same mean number of FITC molecules per cell (Fig. 1f, top), and then added 4m5.3-28z CAR T cells to the labelled tumour cells at a range of effector:target (E:T) ratios. DSPE-PEG_2k_-FITC and DSPE-PEG_3.4k_-FITC elicited robust CAR T cell activation as read out by interferon gamma (IFN-γ) secretion, while the lower MW DSPE-PEG_1k_-FITC was much less effective (Fig. 1f, bottom). Based on its optimal cell membrane insertion and CAR T stimulation properties, we thus focused on DSPE-PEG_2k_-FITC for further studies. DSPE-PEG_2k_-FITC tagging triggered E2-28z CAR T cell activation accompanied by cytokine secretion and efficient killing of B16F10 cells and CT-2A murine glioma cells by FITC CAR T cells (Fig. 1g,h). Thus, with this optimal amph-FITC molecule, diverse cancer cells can be labelled for effective CAR T cell activation and target cell killing.

### Intratumoural administration of amph-FITC labels tumour cells

**Fig. 2:**
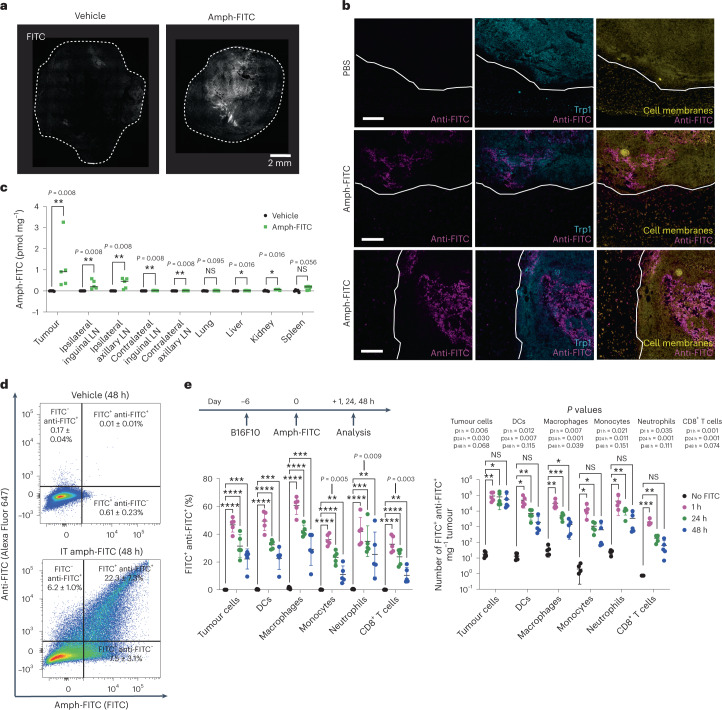
Intratumoural administration of amph-FITC decorates cancer cells and draining LNs with minimal labelling of other tissues. C57BL/6 mice were inoculated with 10^6^ B16F10 tumours in the flank, following by i.t. injection of 10 nmol DSPE-PEG_2k_-FITC when tumours reached 25 mm^2^ in size. **a**, Confocal microscopy of B16F10 tumours 24 h after i.t. amph-FITC injection. Shown is one representative histological image from two tumours analysed. **b**, A total of 10^6^ B16F10 cells were inoculated in C57BL/6 mice (*n* = 4 per group) and injected intratumourally with 10 nmol amph-FITC when tumours were ~25 mm^2^ in size. Two hours later, tumours were isolated with neighbouring connective tissue, cryosectioned and stained with anti-Trp1 antibody to identify melanoma cells, anti-FITC and a cell membrane stain. Shown are representative sections from one PBS control and two amph-FITC-injected tumours. Scale bars, 200 μm. **c**, Biodistribution of amph-FITC 24 h following i.t. injection into B16F10 tumours (*n* = 5 animals per group). **d**, Representative flow cytometry plots of amph-FITC-injected B16 tumours (gated on CD45^−^ cells) stained with anti-FITC to detect surface-exposed antigen. **e**, Immunophenotyping of B16F10 tumours in mice without prior LD at 1, 24 and 48 h following amph-FITC injection, quantifying the proportion of FITC^+^ anti-FITC^+^ double-positive cells (left), and density of FITC^+^ anti-FITC^+^ cells in the tumour (right) (*n* = 5 animals per group). All replicates are biological replicates. *P* values were determined by Mann–Whitney *U* test (**d**) or unpaired Student’s *t*-test (**e**). Shown are mean ± s.d. NS, not significant; *P < 0.05, **P < 0.01, ***P < 0.001, ****P < 0.0001. Source data

We next assessed tumour labelling in vivo following i.t. injection of amph-FITC, using a dose of amph-FITC (10 nmol) that we previously found to be effective for vaccine-boosting CAR T cells^21^. Following a single injection into established B16F10 tumours, amph-FITC labelled large regions of the tumour as revealed by histology (Fig. 2a). To assess potential leakage of amph-FITC outside the tumour into surrounding healthy tissue, we analysed tumour sections and adjacent non-tumour tissue by immunohistochemistry. While i.t. injections did lead to amph-FITC accumulating near the edges of tumours in some instances, we saw very sparse uptake of amph-FITC on cells in the neighbouring tissue (Fig. 2b). Quantification of FITC extracted from the tumour, tumour-draining lymph nodes (TDLNs), and other distal tissues 24 h post injection revealed that amph-FITC remained largely confined to the tumour and draining inguinal and axillary LNs (Fig. 2c). We hypothesized that amph-FITC uptake into the draining LNs could support CAR T cell proliferation and function by decorating LN-resident APCs, as described in our earlier studies employing amph-FITC as a vaccine for CAR T cells^21^.

Flow cytometry analysis showed that FITC was taken up by a large proportion of tumour cells 24 h after injection, and remained accessible at cell surfaces as detected by staining with an anti-FITC antibody (Fig. 2d). As expected, among the many cell types within the tumour, both cancer cells and host cells (that is, infiltrating myeloid cells and T cells) were tagged with FITC (Fig. 2e and Supplementary Fig. 3a), with ~40–60% of each cell type bearing extracellularly exposed FITC after 1 h, and 20–40% of cells still bearing exposed FITC at 24 h (Fig. 2e, left and Supplementary Fig. 4). However, due to their relative abundance, numerically by 24 h the vast majority of cells surface-decorated with FITC were the cancer cells (Fig. 2e, right). As lymphodepletion (LD) commonly used to promote CAR T cell engraftment might affect amph-FITC uptake, we also carried out analysis of FITC cellular distribution in animals that received LD by 5 Gy total body irradiation (TBI) 24 h before amph-FITC injection. LD pre-treatment substantially depleted immune cells and favoured labelling of cancer cells in an even greater proportion (Supplementary Figs. 3b,c and 4). Altogether, these data indicate that i.t. administration effectively restricts amph-FITC availability to the tumour and TDLNs, leading to robust FITC labelling on the surface of cancer cells.

### FITC-specific CAR T cells home to and expand in tumours

**Fig. 3:**
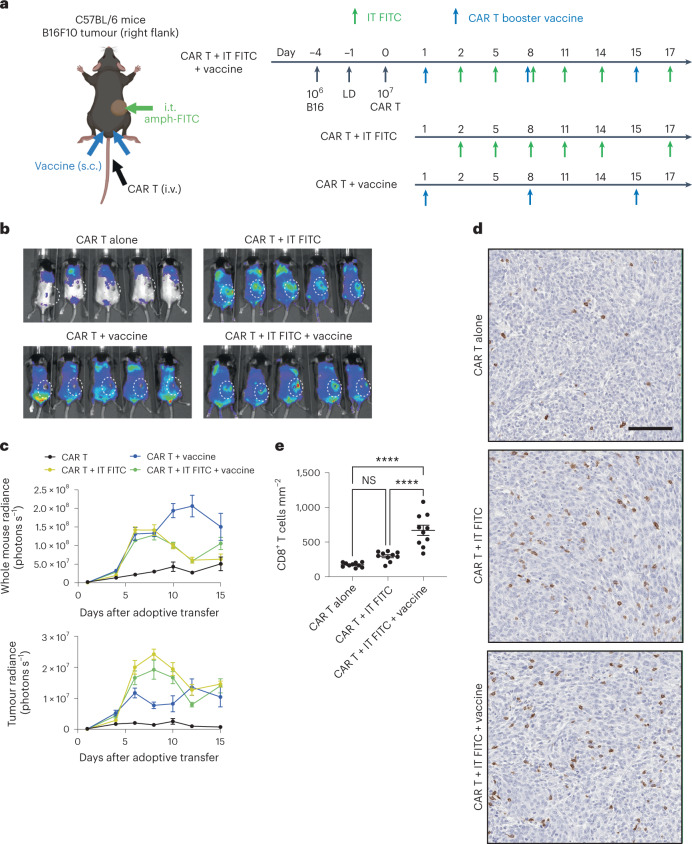
FITC CAR T cells infiltrate into tumours and expand in the presence of amph-FITC. **a**, Schematic and timeline of therapy in B16F10 tumours, including CAR T booster vaccine. Created with Biorender.com. **b**,**c**, Localization of FLuc^+^ E2-m28z FITC CAR T cells in B16F10 tumour-bearing mice on day 8 (**b**) and quantification of whole mouse (top) and tumour (bottom) radiance (**c**) (*n* = 5 animals per group, biological replicates). White dashed circle indicates location of flank tumour. **d**, Representative immunohistochemistry on CT-2A tumours treated with i.t. amph-FITC ± amph-FITC vaccine, staining for CD8, at day 12 post-adoptive transfer. Scale bar, 50 µm. **e**, Quantification of CD8^+^ T cells per mm^2^ per field of view (FOV) in 10 FOV per tumour, *n* = 10 FOVs per condition. Replicates are technical replicates to capture T cell infiltration across many FOVs within the tumour. *P* values were determined by one-way ANOVA with Tukey’s post hoc test. Error bars represent standard error of the mean (**c**) and s.d. (**e**). NS, not significant; ****P < 0.0001. Source data

**Extended Data Fig. 1:**
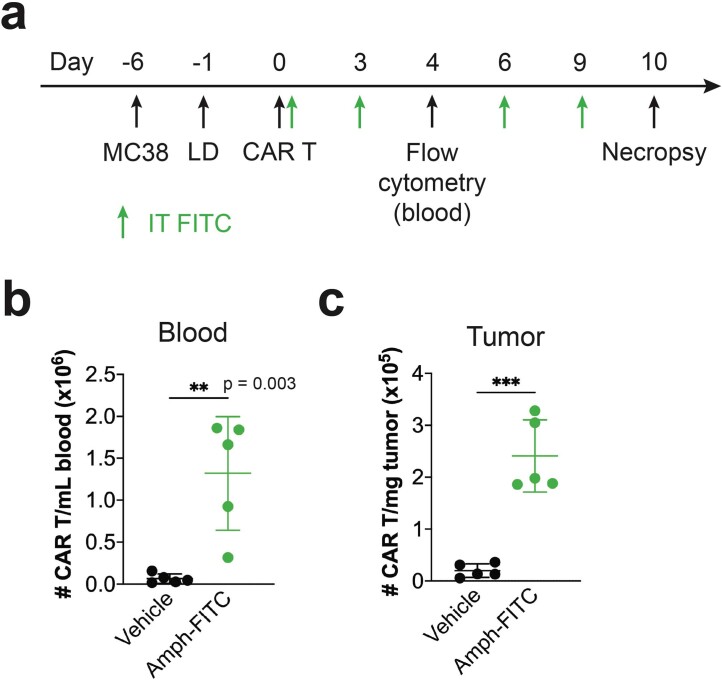
FITC CAR T cells expand in the peripheral blood and tumour with amph-FITC injection. **a** Timeline of therapy in MC38 tumour-bearing C57BL/6 mice. **b, c** CAR T cells in the peripheral blood (**b**, day 4) and tumour (**c**, day 11) (*n* = 5 animals/group). *p* values were determined by unpaired Student’s *t* test. Error bars represent standard deviation. *p < 0.05, **p < 0.01, ***p < 0.001, ****p < 0.0001. Source data

Next, we characterized the ability of FITC-specific CAR T cells to recognize tumours injected with amph-FITC, and tracked CAR T cell expansion and tumour homing via bioluminescence imaging. Mice bearing B16F10 tumours were lymphodepleted by TBI, followed by adoptive transfer of firefly luciferase-expressing E2-28z CAR T cells. Beginning 2 days later, amph-FITC was administered intratumourally and every 3 days thereafter to re-tag any tumour cells that had not yet been killed by CAR T cells (Fig. 3a, CAR T + IT FITC). We chose to give the first dose of i.t. amph-FITC after CAR T administration to maximize the time for CAR T cells to encounter tumour cells decorated with a high level of the FITC ligand. To further support CAR T cell attack on FITC-painted tumours, in a separate group we also tested the effect of CAR T cell transfer (± i.t. amph-FITC) combined with a once-weekly bilateral subcutaneous injection of amph-FITC together with the stimulator of interferon genes (STING) agonist cyclic-di-GMP adjuvant as a vaccine boost. This injection targets amph-FITC to DCs in draining LNs and acts as a vaccine for the CAR T cells, triggering CAR T cell expansion and increased functionality as we previously reported^21^. Bioluminescence imaging of CAR T cells transferred into mice in the absence of amph-FITC treatment revealed a low degree of E2-28z CAR T cell expansion over 14 days without substantial infiltration of tumours (Fig. 3b,c). Administration of i.t. amph-FITC or amph-FITC vaccination individually triggered CAR T cell expansion, but amph-FITC vaccination alone triggered more prominent CAR T cell accumulation near the vaccine injection site near the base of the tail rather than in the tumour. By contrast, i.t. amph-FITC led to comparable total CAR T cell expansion over the first 7 days, but greater accumulation at the tumour site (Fig. 3b,c). Addition of the vaccine boost to i.t. amph-FITC treatment did not have a notable impact on overall T cell expansion or accumulation at the tumour site. We repeated this experiment treating CT-2A tumours, and analysis of treated tumours at day 12 revealed substantial T cell infiltration throughout the tumour (most likely by CAR T cells due to the temporal proximity to LD) (Fig. 3d,e). We also analysed E2-28z CAR T cell infiltration of MC38 colon carcinomas, and found increases in the abundance of CAR T cells in both the peripheral blood and in the tumour following i.t. amph-FITC treatment (Extended Data Fig. 1a–c). Thus, i.t. amph-FITC treatment both with and without supporting amph-FITC vaccination leads to significant expansion of the CAR T cell population and infiltration of tumours by E2-28z CAR T cells.

### Antitumour activity of amph-FITC/FITC-specific CAR T cell therapy

**Fig. 4:**
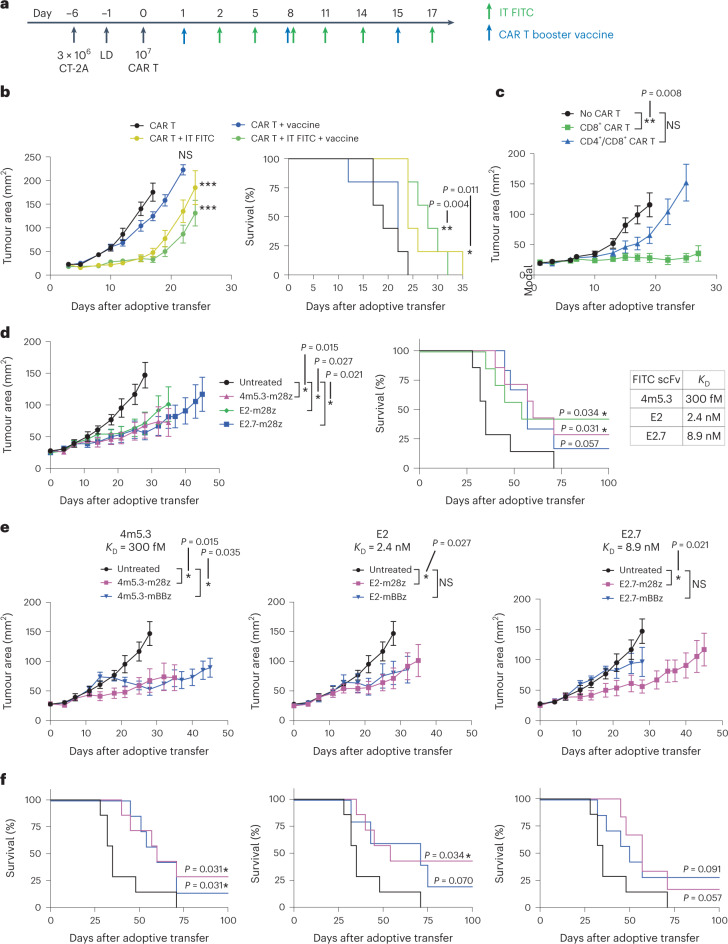
Murine FITC CAR T cells combined with i.t. amph-FITC have therapeutic activity in models of melanoma and glioma. **a**, Schematic and timeline of therapy for CT-2A tumour therapy. **b**, Tumour growth and overall survival of C57BL/6 mice (*n* = 5 animals per group) bearing B16F10 tumours treated with E2-28z CAR T cells as in Fig. 3a with indicated combinations. **c**, Tumour growth of CT-2A tumour-bearing C57BL/6 mice (*n* = 5 animals per group) treated with amph-FITC, FITC CAR T cells and vaccine composed of only CD8^+^ T cells or a combination of CD4^+^ and CD8^+^ T cells. **d**, Tumour growth and survival following treatment of CT-2A tumour-bearing mice (*n* = 5 animals per group) with CARs with low (E2.7), medium (E2) and high (4m5.3) affinities for FITC, including i.t. amph-FITC and vaccine. **e**,**f**, Tumour growth (**e**) and survival (**f**) of CT-2A tumour-bearing mice treated with CARs bearing CD28 or 4-1BB co-stimulatory domains across a range of binding affinities in combination with IT amph-FITC and amph-FITC vaccination (*n* = 5 animals per group). Error bars represent standard error of the mean. All replicates are biological replicates. *P* values were determined by two-way ANOVA (tumour growth curves) and log-rank (Mantel–Cox) test (survival curves). NS, not significant; *P < 0.05, **P < 0.01, ***P < 0.001. Source data

**Extended Data Fig. 2:**
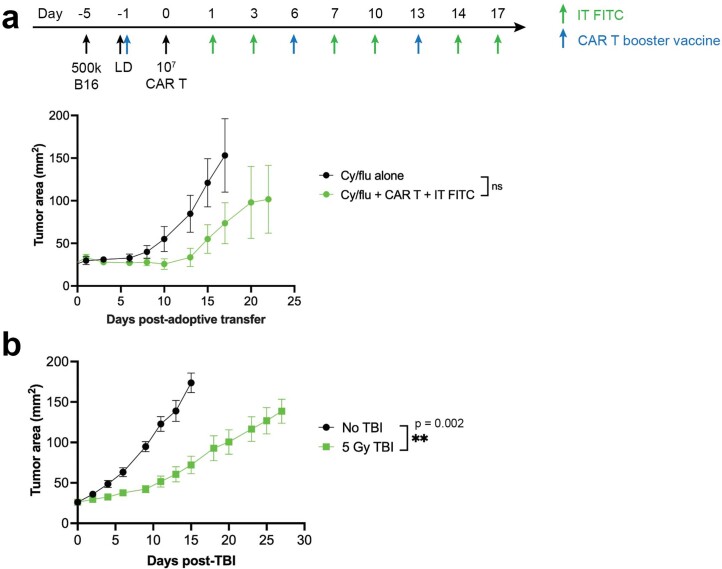
Chemotherapy-based vs. irradiation-based lymphodepletion. **a** Groups of C57BL/6 mice bearing B16F10 tumours (*n* = 5 animals/group) were lymphodepleted with 250 mg/kg cyclophosphamide and 50 mg/kg fludarabine administered one day before adoptive cell transfer, followed by treatment with intratumoural amph-FITC and amph-FITC vaccine boosting as indicated. **b** Comparison of tumour growth in mice left untreated (*n* = 7) vs. animals receiving lymphodepleting regimen of 5 Gy TBI (*n* = 8) seven days after being inoculated with 3×10^6^ CT-2A cells. *p* values were determined by two-way ANOVA. Error bars represent standard error of the mean. ns, not significant; **p < 0.01. Source data

We then evaluated the therapeutic impact of FITC-targeting CAR T cell transfer combined with i.t. amph-FITC and amph-FITC vaccination in the B16F10 and CT-2A tumour models, following the experimental timelines outline in Fig. 3a and Fig. 4a, respectively. We first tested treatment of the highly aggressive, poorly immunogenic B16F10 model, and evaluated treatment with or without the inclusion of amph-FITC vaccine boosting. As shown in Fig. 4b, E2-28z CAR T cells redirected by i.t. amph-FITC halted tumour progression for ~2 weeks and extended survival; addition of amph-FITC vaccine boosting exhibited a trend towards slightly better tumour regression and extension of survival, increasing median survival by 3 days. To determine whether the method of LD impacts response to this therapy, we also tested treatment using the clinical chemotherapy LD regimen of cyclophosphamide and fludarabine before CAR T cell transfer, and observed similar antitumour activity (Extended Data Fig. 2a).

We next turned to the CT-2A tumour model, as a slowly progressing tumour more amenable to immunotherapy treatment, and sought to dissect factors governing the therapeutic efficacy of FITC CAR T treatment. First, we tested the effect of the CD4/CD8 composition in the CAR T cell product. Adoptive transfer of exclusively CD8^+^ E2-28z CAR T cells combined with i.t. amph-FITC and vaccination halted progression of CT-2A tumours for 3 weeks (Fig. 4c). Although LD alone delayed tumour progression (Extended Data Fig. 2b), this effect was much weaker than the response elicited by combining adoptive transfer with i.t. amph-FITC. Interestingly, in contrast to evidence supporting an important role for CD4^+^ CAR T cells in humanized mouse models of mesothelioma or lymphoma^38,39^, transfer of an equivalent number of murine CAR T cells prepared with a ~1:1 CD8^+^:CD4^+^ ratio was substantially less effective (Fig. 4c).

Given the wealth of literature suggesting important effects of CAR affinity and co-stimulatory domain selection on the outcome of traditional CAR T therapy^40–49^, we next assessed the impact of these CAR design parameters on the efficacy of amph-FITC-redirected CAR T treatment. To modulate CAR affinity, we compared the efficacy of CAR T cells bearing the E2 FITC-specific scFv (*K*_D_ = 2.4 nM) with CARs prepared from a very-high-affinity FITC scFv, 4m5.3 (*K*_D_ = 300 fM), or the lower-affinity scFv E2.7 (*K*_D_ = 8.9 nM), which differs from wild-type (WT) E2 by a single amino acid substitution. Each of these three scFv variant CARs was prepared with either a CD28 or tumour necrosis factor ligand superfamily member 9 (TNFSF9, hereafter referred to as 4-1BB) co-stimulatory domain to further interrogate the role of co-stimulatory signalling. As shown in Supplementary Fig. 5, murine CD8^+^ T cells showed efficient transduction and expression of each of these CARs, though the CD28-based CARs expressed at ~10-fold higher levels than the 4-1BB CARs. In tandem with i.t. amph-FITC + FITC vaccination, all of these FITC-specific CAR T cells displayed similar levels of therapeutic efficacy in treating C2TA tumours and extending animal survival, with the exception of the E2.7-BBz CAR that was slightly less effective (Fig. 4d–f). However, CARs with CD28 co-stimulatory domains demonstrated earlier tumour growth control compared with those with 4-1BB co-stimulatory domains, independent of the affinity of the scFv (Fig. 4e). The most effective E2-28z CAR elicited complete responses in 40% of animals. We opted to focus on this CAR for subsequent studies, based on its therapeutic efficacy and superior expression.

**Extended Data Fig. 3:**
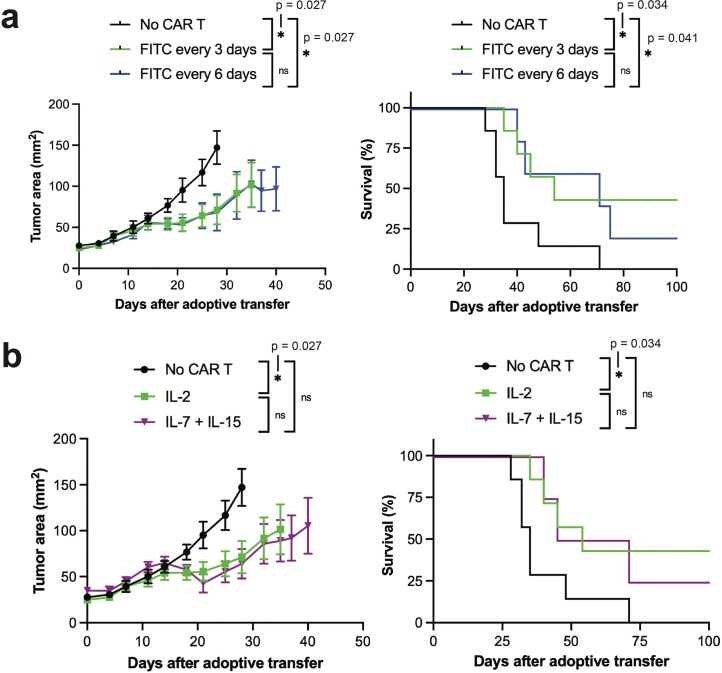
Optimization of FITC CAR T and amph-FITC therapy. **a** Comparison of tumour growth (left) and survival (right) in CT-2A tumour-bearing mice treated with CAR T cells followed by intratumoural amph-FITC every 3 or 6 days (*n* = 5 animals/group). **b** Comparison of tumour growth (left) and survival (right) in CT-2A tumour-bearing mice treated with FITC CAR T cells prepared in mIL-2 or a combination of mIL-7 and mIL-15 (*n* = 5 animals/group). *P* values were determined by two-way ANOVA (tumour growth curves) and log-rank (Mantel-Cox) test (survival curves). Error bars represent standard deviation. n.s., not significant; *p < 0.05, **p < 0.01, ***p < 0.001, ****p < 0.0001. Source data

We finally carried out experiments aiming to optimize the dosing of amph-FITC and preparation of maximally effective CAR T cells in this treatment paradigm. We first assessed the importance of dosing frequency, since i.t. dosing every few days for several weeks may only be clinically feasible for superficial lesions in diseases such as melanoma or head and neck cancer. Interestingly, reducing injection frequency to once every 6 days did not reduce the therapeutic effect (Extended Data Fig. 3a). Finally, ex vivo culture of CAR T cells in IL-7 and IL-15 rather than traditional IL-2 is also known to favour the central memory (T_CM_) phenotype and increase tumour control^50–52^. However, FITC CAR T cells expanded in IL-7 and IL-15 rather than IL-2 did not demonstrate enhanced tumour control (Extended Data Fig. 3b). Thus, we finalized our therapy to use E2-28z CAR T cells expanded in IL-2 for further studies.

### Amph-FITC and FITC CAR T therapy is well tolerated with minimal toxicity

**Extended Data Fig. 4:**
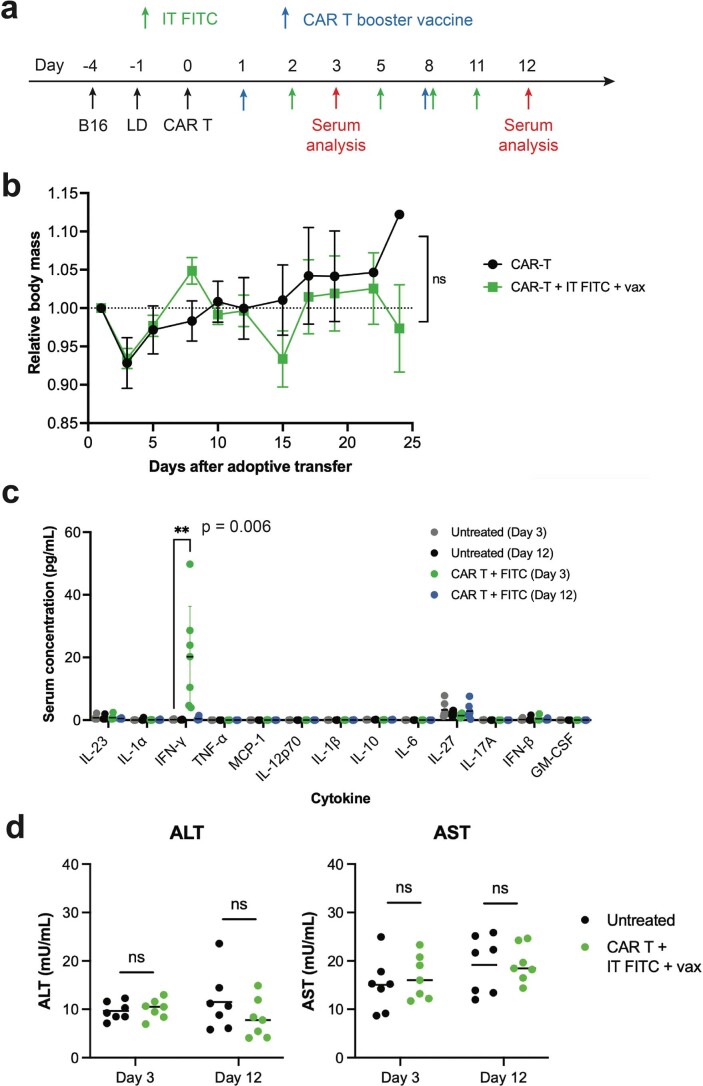
FITC CAR T and amph-FITC therapy elicit minimal toxicity. **a** Timeline of therapy in CT-2A tumour-bearing C57BL/6 mice. **b** Body weight of B16F10 tumour-bearing C57BL/6 mice over the course of therapy (*n* = 5 animals/group). **c, d** Quantification of serum cytokines (**c***, n* = 5 animals/group) and serum ALT and AST (**d**, *n* = 7 animals/group) at 3 and 12 days post-adoptive transfer. *P* values were determined by two-way ANOVA (body weight curves) or unpaired Student’s *t* test. Error bars represent standard error of the mean (**b**) and standard deviation (**c**). *p < 0.05, **p < 0.01, ***p < 0.001, ****p < 0.0001. Source data

A chief concern with the chemical delivery of a CAR T ligand is the potential for dissemination of amph-FITC to trigger CAR T cell attack on healthy tissues. We found that local i.t. administration of amph-FITC minimally labels normal tissues (Fig. 2b), and we did not detect obvious inflammation of the skin surrounding treated tumours during treatment, suggesting that the CAR T cell activity remains primarily i.t. However, the finding that E2-28z CAR T cells expanded in the peripheral blood following i.t. amph-FITC treatment (Extended Data Fig. 1b) prompted us to evaluate potential systemic toxicities. In therapy studies, mice receiving CAR T cells together with i.t. amph-FITC and FITC vaccination showed no weight loss and gained mass over time indistinguishable from untreated control animals (Extended Data Fig. 4a,b). We measured serum cytokines 24 h after amph-FITC injection on day 3 and day 12 following E2-28z CAR T cell transfer in the CT-2A tumour model, and saw no elevation of inflammatory cytokines, with the exception of a low level of IFN-γ at day 3 (Extended Data Fig. 4c). We also failed to detect elevation of the liver enzymes alanine transaminase (ALT) or aspartate transaminase (AST) at corresponding timepoints, indicating a lack of liver toxicity (Extended Data Fig. 4d). Thus, therapy with i.t. amph-FITC and FITC-specific CAR T cells appears to be safe and well tolerated.

### CAR T cell-mediated cytotoxicity stimulates an endogenous antitumour T cell response

**Fig. 5:**
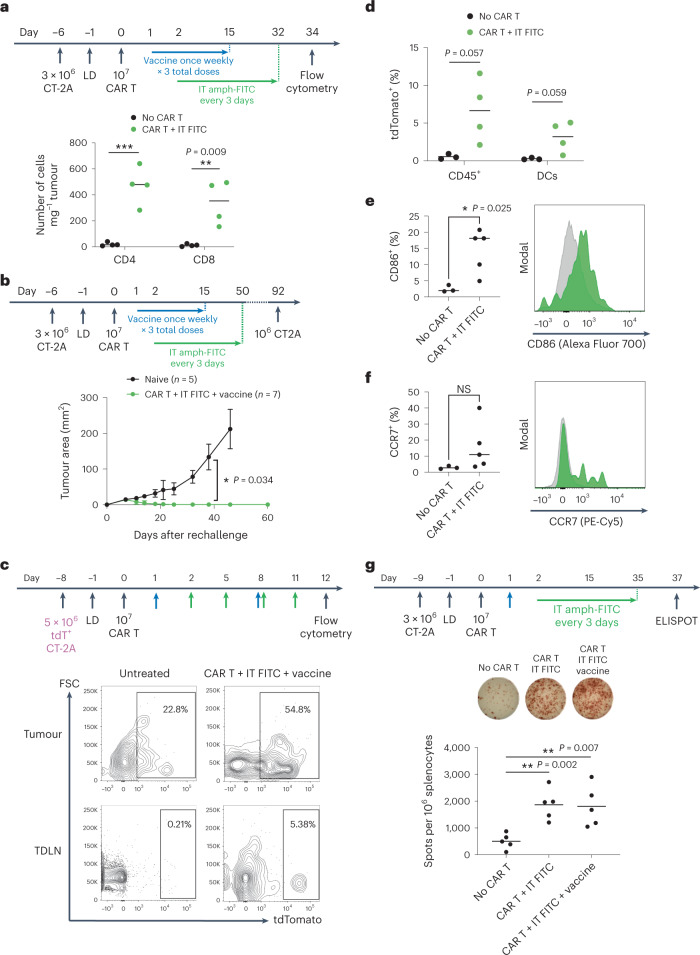
Amph-FITC therapy induces epitope spreading to elicit an endogenous antitumour T cell response. **a**, Flow cytometry on treated CT-2A tumours on day 34 after adoptive transfer of CD45.2^+^ FITC CAR T cells, quantifying CD4^+^ versus CD8^+^ CD45.1^+^ tumour-infiltrating host T cells (*n* = 4 animals per group). **b**, CT-2A tumour-bearing mice previously cured with CAR T and amph-FITC therapy were rechallenged versus naïve mice with 10^6^ CT-2A cancer cells on the opposite flank at day 92 following adoptive transfer (*n* = 5 animals per group). **c**,**d**, Representative flow plots of DCs (**c**) in tumours and TDLN of mice bearing tdTomato^+^ CT-2A tumours at 12 days post-adoptive transfer and quantification (**d**) of tdTomato^+^ DCs in TDLN (*n* = 3 animals per group for no CAR T group, *n* = 4 for CAR T + i.t. FITC group). **e**,**f**, Expression of activation markers CD86 (**e**) and CCR7 (**f**) in TDLN DCs at 12 days post-adoptive transfer with representative histograms (*n* = 3 animals per group for no CAR T group, *n* = 5 for CAR T + i.t. FITC group). **g**, ELISPOT on spleens of CT-2A tumour-bearing mice on day 37 post adoptive transfer (*n* = 5 animals per group). Splenocytes were stimulated with irradiated CT-2A cancer cells. All replicates are biological replicates. *P* values were determined by unpaired Student’s *t*-test. Error bars represent s.d. NS, not significant; *P < 0.05, **P < 0.01. Source data

We hypothesized that by redirecting CAR T cells against injected lesions, tumour antigens could be released in a favourable pro-inflammatory microenvironment that would drive endogenous T cell priming and induction of a systemic antitumour immune response. Although we employed TBI to promote CAR T cell engraftment at the start of treatment, it is known that lymphocytes rapidly recover following LD^53^, and other studies have reported evidence of endogenous immune responses primed by CAR T cell therapy despite the use of lymphodepleting regimens^21,25^. We first evaluated infiltration of host CD45.2^+^ CD4^+^ and CD8^+^ T cells into CT-2A tumours treated with i.t. amph-FITC and CD45.1^+^ CAR T cells. While untreated tumours showed very low levels of tumour-infiltrating lymphocytes, amph-FITC/CAR T treatment elicited robust infiltration by CD45.2^+^ host T cells (Fig. 5a). To assess potential induction of endogenous T cell memory by amph-FITC/CAR T treatment, we rechallenged mice that had rejected CT-2A tumours in response to amph-FITC/E2-28z CAR T therapy with tumour cells on the opposite flank. While control naïve mice inoculated with CT-2A cells showed steady tumour progression, all of the mice cured by amph-FITC/CAR T treatment rejected the rechallenge, despite the absence of FITC in the new tumour (Fig. 5b), suggesting the induction of protective endogenous T cell memory.

A key step for priming of endogenous T cell responses is acquisition of tumour antigen by dendritic cells (DCs). To determine if amph-FITC/CAR T therapy promoted DC uptake of tumour cell debris, we treated tdTomato^+^ CT-2A tumours and found that immune cells, most notably DCs in both the tumour and tumour-draining LN, became tdTomato^+^, suggesting uptake of tumour antigens (Fig. 5c,d). Additionally, more DCs in the TDLN expressed activation markers CD86 and CCR7 (Fig. 5e,f). Hence, redirection of CAR T cells against tumours through amph-FITC decoration promotes antigen delivery to and activation of professional APCs that govern endogenous T cell priming. Finally, we directly assayed for the development of tumour-specific endogenous T cells following amph-FITC/E2-28z CAR T cell treatment by restimulating splenocytes from amph-FITC/CAR T-treated mice ex vivo with irradiated CT-2A tumour cells. As shown in Fig. 5g, restimulation of splenocytes from treated animals with CT-2A cells confirmed that amph-FITC/CAR T treatment amplified tumour-specific endogenous T cell responses relative to untreated tumour-bearing mice, though addition of amph-FITC vaccine boosting did not appear to provide additional benefit over i.t. amph-FITC administration alone.

### Amph-FITC-mediated CAR T cell stimulation triggers a systemic antitumour immune response

**Fig. 6:**
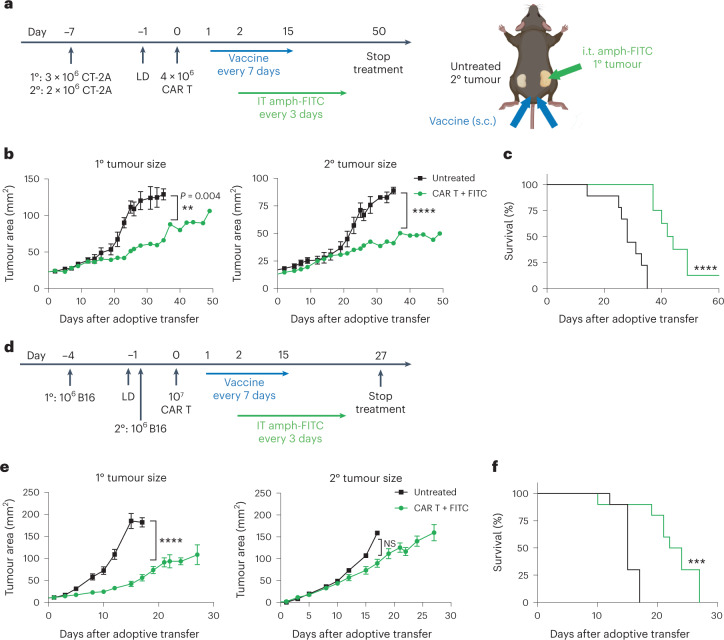
Local amph-FITC redirection of CAR T cells leads to a systemic antitumour immune response. **a**–**c**, C57BL/6 mice (untreated *n* = 10, treated *n* = 8) were inoculated with CT-2A tumour cells on opposite flanks, then treated with FITC-CAR T cells, amph-FITC vaccination and i.t. amph-FITC only in the 1° lesion. Shown are the timeline and schematic of the treatment (**a**), mean tumour size (**b**) and overall survival (**c**) over time. Created with Biorender.com. **d**–**f**, C57BL/6 mice (*n* = 10 per group) were inoculated with B16F10 tumour cells on opposite flanks, then treated with FITC-CAR T cells, amph-FITC vaccination and i.t. amph-FITC only in the 1° lesion. Shown are the timeline of the treatment (**d**), mean tumour size (**e**) and overall survival (**f**) over time. Error bars represent standard error of the mean. All replicates are biological replicates. *P* values were determined by two-way ANOVA (tumour growth curves) and log-rank (Mantel–Cox) test (survival curves). NS, not significant; **P < 0.01; ***P < 0.001, ****P < 0.0001. Source data

Encouraged by the evidence that i.t. amph-FITC/CAR T treatment elicits endogenous T cell priming, we next assessed whether this localized therapy could trigger systemic antitumour immune responses. To this end, we implanted CT-2A tumours bilaterally in the flanks of mice, and treated only the right flank tumour by administration of i.t. amph-FITC (in combination with amph-FITC vaccination, Fig. 6a). Strikingly, both the injected (1°) and non-injected (2°) tumours showed substantially slowed progression, and overall survival of the treated animals was significantly improved (Fig. 6b,c). We then tested the ability of local amph-FITC delivery to elicit systemic immunity against B16F10 melanomas; for this more aggressive model the un-injected secondary tumour was inoculated several days after the to-be-treated primary tumour, to allow the endogenous T cell response time to develop before rampant distal tumour outgrowth (Fig. 6d). Injection of only the primary tumour led to slowed growth of the treated tumour and a trend towards slowed progression of the untreated lesions, in addition to significant increases in overall survival over untreated animals (Fig. 6e,f). Thus, the endogenous T cell response initiated by local redirection of CAR T cells with amph-FITC injection leads to immune attack of distal untreated tumours.

### Amph-FITC tagging enables human CAR T cells to target xenograft tumours

**Fig. 7:**
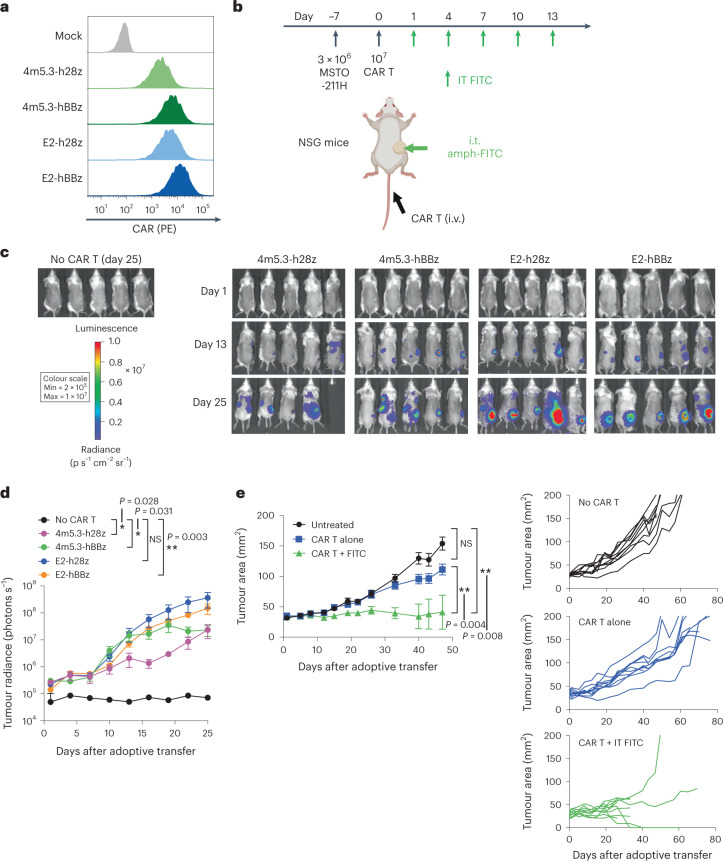
Amph-FITC CAR T cell therapy is efficacious when translated to human FITC CAR T cells in human solid tumour xenografts. **a**, Expression of humanized FITC CARs. **b**, Schematic and timeline of therapy in NSG mice. Created with Biorender.com. **c**,**d**, Bioluminescence imaging (**c**) and quantification (**d**) of FLuc^+^ CAR T cell trafficking in NSG mice (*n* = 5 animals per group). **e**, MSTO-211H tumour growth of NSG mice following treatment with E2-hBBz CAR T cells (*n* = 10 animals per group). All replicates were biological replicates. *P* values were determined by two-way ANOVA. Error bars represent standard error of the mean. **P < 0.01; ***P < 0.001. NS, not significant. Source data

**Extended Data Fig. 5:**
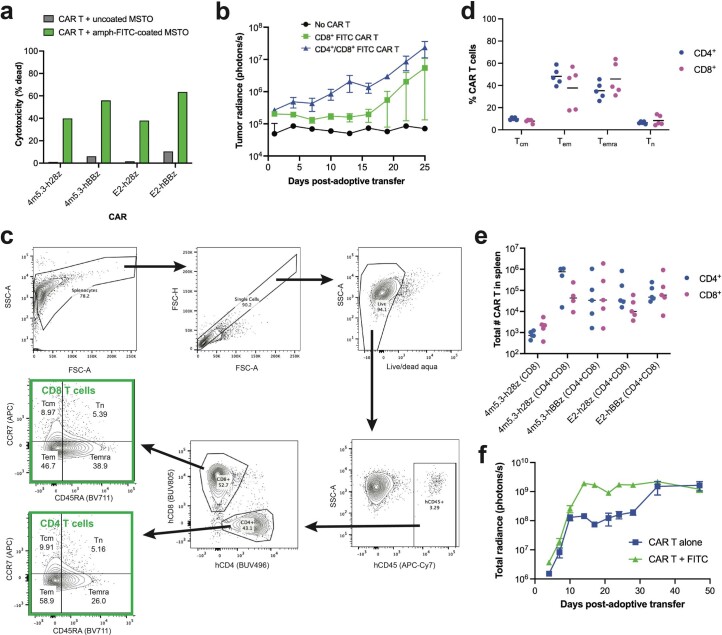
Expansion and tumour localization of human FITC CAR T cells in NSG mice. **a** *In vitro* cytotoxicity of FITC CAR T cells with amph-FITC^+^ MSTO-211H human mesothelioma cancer cells at 1:1 ET ratio. **b** Comparison of luciferase signal from fLuc^+^ CAR T cells over time in mice treated with only CD8^+^ 4m5.3-h28z CAR T cells or a combination of CD4^+^ and CD8^+^ CAR T cells (*n* = 5 animals/group). **c, d** Gating strategy for immunophenotyping human CAR T cells (**c**) and phenotypic composition of CAR T cells recovered from spleens of NSG mice 23 days post-adoptive transfer (**d**, *n* = 5 animals/group). **e** Quantification of CD4^+^ and CD8^+^ CAR T cells in the spleen on day 40 post-adoptive transfer (*n* = 5 animals/group). **f** Whole mouse radiance over time as a surrogate of CAR T cell expansion (*n* = 5 animals/group). Error bars represent standard error of the mean. Source data

We focused our initial studies in immunocompetent mouse models using murine CAR T cells to enable analysis of antigen spreading and endogenous T cell immunity, but the ability of amph-FITC tagging to effectively redirect human CAR T cells against human cancer cells was also important to evaluate. To this end, we engineered FITC-specific CARs that were highly expressed in human T cells (Fig. 7a). In vitro, human CAR T cells prepared with either high-affinity 4m5.3 or lower-affinity E2 scFv-based CARs exhibited potent cytotoxicity against amph-FITC-tagged MSTO-211H human mesothelioma tumour cells, irrespective of the use of CD28 or 4-1BB co-stimulatory domains (Extended Data Fig. 5a). Thus, human CAR T cells also effectively recognize amph-FITC-coated cancer cells.

In most of our syngeneic murine tumour treatment studies, we administered i.t. amph-FITC alongside subcutaneously (s.c.) administered amph-FITC (with adjuvant) that is efficiently taken up in draining LNs as a vaccine booster^21^. However, because lymphatic vessels and peripheral LNs are defective in the immunodeficient NOD.Cg-*Prkdc*^*scid*^*IL2rg*^*tm1Wjl*^*/SzJ* (NSG) mice used for human CAR T cell evaluation^54^, we were restricted to stimulating FITC-specific CAR T cells through i.t. amph-FITC. To test the in vivo activity of CAR T cells responding to amph-FITC-coated tumours, we inoculated MSTO-211H tumours in NSG mice, adoptively transferred luciferase-expressing 4m5.3 or E2 CAR T cells with CD28 or 4-1BB co-stimulatory domains 7 days later, and then treated animals with repeated doses of i.t. amph-FITC (Fig. 7b). In contrast to murine CAR T cells, where transfer of a pure CD8^+^ CAR T cell population had superior therapeutic efficacy to a mixed population of CD4^+^ and CD8^+^ CAR T cells, we found that human CAR T cells proliferated more robustly as a mixed CD4^+^ and CD8^+^ population following amph-FITC administration (Extended Data Fig. 5b), consistent with previous studies^38,39^. Therefore, we proceeded with a mixed CD4/CD8 population for human T cell experiments. Analysis of the CAR T cells post adoptive transfer showed that these human CAR T cells were composed of a mixture of effector memory (T_em_) and effector memory cells re-expressing CD45RA (T_emra_) phenotypes (Extended Data Fig. 5c,d).

On treating tumours as in Fig. 7b, all of the CAR T cells showed some level of accumulation in tumours between day 13 and day 25, but E2-h28z and E2-hBBz CAR T cells showed the most accumulation at the injected tumour sites (Fig. 7c,d). Enumeration of CAR T cells in the spleen on day 40 also showed persistence of all of the CAR T cells systemically, with the exception of the 4m5.3-h28z CAR T cells infused as a pure CD8^+^ population (Extended Data Fig. 5e). Due to its higher expression, superior in vitro tumour cytotoxicity, and robust accumulation in tumours, we proceeded forward with the E2-hBBz CAR construct for human T cell therapy studies. In combination with i.t. amph-FITC, E2-hBBz CAR T cells led to complete tumour rejection in ~20% of mice and tumour growth was halted in another 60% of the cohort (Fig. 7e). No overt toxicities in terms of animal weight loss or alterations in behaviour were noted during treatment until late timepoints when both treated and untreated groups began showing symptoms of graft-versus-host disease (GVHD), a known limitation of the NSG mouse model. Notably, the 13.3-fold relative expansion of CAR T cells induced by amph-FITC treatment (Extended Data Fig. 5f) exacerbated GVHD in this group, leading to early death of a number of the animals that were responding to treatment (Fig. 7e). Hence, amph-FITC injection appears a promising strategy to redirect human CAR T cells against solid tumours.

## Discussion

So far, the translation of CAR T cell therapy to solid tumours has been limited by the imperfect nature of tumour antigens, with heterogeneous antigen expression and poor tumour specificity leading to treatment resistance and toxicity to healthy tissues, respectively^8–11^. Here we describe a therapy employing a phospholipid–polymer conjugate that, when delivered locally via i.t. injection, labels cells within the tumour with a small molecule antigen, FITC, and tags cancer cells for destruction by FITC-specific CAR T cells. This treatment prolonged survival in multiple syngeneic murine solid tumours and also elicited tumour regression in a human solid-tumour xenograft model. Importantly, amph-FITC-directed CAR T cytotoxicity triggered the release of tumour antigens and the activation of DCs, leading to priming of endogenous antitumour T cells. This endogenous immunity was sufficient to protect from tumour rechallenge, and provides a mechanism for endogenous immune attack against non-injected lesions.

This strategy enables control of tumour targeting via local i.t. administration of amph-FITC, ensuring that CAR T cells can effectively recognize cancer cells while limiting on-target off-tumour toxicity at distal healthy tissues. Prior studies have used FITC-specific CAR T cells and redirected these cells against tumours by linking FITC to tumour-targeted antibodies^55–57^ or metabolites such as folate^58–60^. But the key limitation of such approaches is that we lack suitable target antigens on the majority of solid cancers for the generation of such switches. FITC-CAR T cells have been redirected against tumours using anti-CD19 or anti-CD20 antibodies coupled to FITC^55,56^, but these antigens are expressed on only a subset of leukaemias and lymphomas, and we already have effective CAR T cell products approved in this setting. Multiple studies have redirected FITC-CAR T cells using the HER2-targeting antibody trastuzumab coupled to FITC^55,57^, but trastuzumab-based CAR T cells have been shown in the clinic to have the potential for lethal toxicity in patients due to low-level expression of HER2 on lung epithelial cells^9^. This is a general concern with tumour-associated antigens such as HER2, EGFR and folate receptor. Despite intensive efforts in the CAR T cell and antibody therapeutic fields, so far identification of broadly targetable and truly tumour-specific cell surface antigens has largely failed. Moreover, even if targeting ligands such as FITC-conjugated antibodies were injected intratumourally as performed here to overcome these concerns, they face the additional challenge that tumour antigen expression can vary widely patient to patient even within a given tumour type (such as EGFRvIII in glioblastoma^11^).

Here we show an entirely tumour antigen-agnostic approach that does not rely on the identification of cancer cell-specific surface antigens. Furthermore, membrane insertion by amph-FITC should enable CAR T cell redirection against tumours uniformly across patients and tumour types, addressing the challenge of antigen-expression heterogeneity. Although cancer cells are capable of developing resistance to adoptive cell therapy targeting natively expressed proteins by mutation or downregulation of the target antigen, we expect such mechanisms of resistance to be much more challenging with CAR-ligand membrane insertion. As we have demonstrated previously^21^, the process of amph-ligand tagging is highly modular; although this study has been executed with FITC as a model antigen, FITC could be exchanged for a range of other ligands, including but not limited to a protein or peptide antigen, or other small molecules. The amph-FITC ligand is a simple and well-defined molecular entity readily amenable for GMP manufacturing. Ligand-specific CAR T cells can be engineered and expanded using existing workflows, without modification to viral vector production or ex vivo T cell culture. The lipid-polymer DSPE-PEG is a central component of approved drugs such as Doxil^61^, with an established safety profile. Furthermore, as necessary, combination therapy with an amph-ligand vaccine would be seamless, requiring only the addition of a suitable vaccine adjuvant, such as the STING agonist used here.

Evidence is emerging for the ability of CAR T cell-mediated cytotoxicity to elicit antigen spreading in syngeneic murine solid tumour models^21–25^, even when LD regimens are employed before adoptive transfer. Although evidence in patients has been more limited, the induction of novel antibody responses following CAR T cell therapy has been reported in pancreatic cancer^62^, and a case report of a patient treated with HER2 CAR T cells for metastatic rhabdomyosarcoma documented oligoclonal expansion of endogenous T cells^63^, suggesting antigen spreading. Here we found that CAR T recognition of membrane-inserted amph-ligand elicited significant expansions in the endogenous antitumour T cell response, which protected cured mice from rechallenge with parental cancer cells in the absence of the CAR ligand and induced antitumour activity against distal lesions.

A potential drawback of this approach is the need for local injection of amph-ligand. Although this may limit the application of the therapy to accessible tumours, i.t. immunotherapies are becoming more common, with an exponential increase in the corresponding number of clinical trials^64,65^. With the development of additional i.t. therapies, the technologies used for administration of these agents (such as image-guided injections) are also being developed and applied more widely^66^. Whereas visceral tumours are accessible for injection, repeated injections may be less feasible, and we are actively investigating the minimum number of injections necessary to induce tumour regression by amph-FITC/CAR T therapy, and anticipate that as few as two to three injections may be sufficient. We did find that treatment once every 6 days was equivalently effective to dosing every 3 days (Extended Data Fig. 3a). Notably, the US Food and Drug Administration-approved i.t. oncolytic virus immunotherapy talimogene laherparepvec is administered every 2 weeks for at least 6 months following initial dosing.

An additional concern is that amph-ligand decoration will decorate all i.t. cell populations and not just cancer cells. We showed that LD pre-treatments commonly used before CAR T cell transfer already remove the majority of i.t. immune cells, lowering the concern over CAR T cell redirection against host immune-cell infiltrates. Furthermore, i.t. cell populations such as tumour-associated macrophages, myeloid cells and cancer-associated fibroblasts are highly immunosuppressive and tumour promoting, and their elimination has been shown in many other studies to drive the antitumour immune response^67–71^. Hence, amph-FITC decoration and CAR T-mediated killing of these i.t. host cell populations, rather than representing a toxicity, would probably promote tumour rejection.

In summary, we have shown an entirely tumour-agnostic universal CAR T cell therapy based on the membrane insertion of intratumourally administered amph-ligands. This approach exhibited potent antitumour activity against multiple tumour types and in immunocompetent hosts. Strikingly, amph-ligand-directed CAR T cell therapy also induced an endogenous T cell response. Although CAR T cell therapy has had impressive efficacy in targeting leukaemias and lymphomas expressing ubiquitous antigens such as CD19, the administration of amph-CAR ligands provides a strategy for the universal treatment of solid tumours where tumour antigen selection is more problematic, greatly expanding the patient population that might benefit from adoptive cell therapy.

## Methods

### Materials

DSPE-PEG-FITC (PEG MW 1, 2, 3.4 and 5 kDa) was purchased from Creative PEGworks (cat. no. PLS-9926 to 9929). DSPE-FITC and DSPE-PEG_10k_-FITC were purchased from Nanosoft Polymers (cat. no. 10805) and Nanocs (cat. no. PG2-DSFC-10k), respectively. Cyclic di-GMP was purchased from Invivogen.

### Cell lines

Phoenix-ECO, B16F10 and MSTO-211H cells were purchased from ATCC. 293T cells were purchased from Clontech. MC38 and CT-2A-Luc cells were kindly provided by Dr Dane Wittrup from the Massachusetts Institute of Technology (MIT) and Dr Thomas Seyfried from Boston College, respectively. CT-2A and MSTO-211H cells were lentivirally transduced to stably express murine EGFRvIII and tdTomato or human mesothelin, respectively, with selection using puromycin and sorting on a BD FACSAria III Cell Sorter. B16F10, CT-2A, MC38, 293T and Phoenix-ECO cells were cultured in complete Dulbecco’s modified Eagle’s medium (Cytiva; supplemented with 10% foetal bovine serum, 100 U ml^−1^ penicillin and 100 mg ml^−1^ streptomycin). MSTO-211H cells were cultured in complete Roswell Park Memorial Institute (RPMI) medium (Cytiva).

### Construct design

All murine CARs contained the CD8α signal peptide (MALPVTALLLPLALLLHAARP) followed by a Myc-tag (EQKLISEEDL, for analysis of cell surface expression) and a FITC-specific scFv derived from monoclonal antibody clone 4m5.3 (ref. ^36^), E2 (ref. ^37^) or E2 with a His-H58-Ala mutation^72^ (‘E2.7’). Extracellular domains were fused to the CD8α hinge and transmembrane domains, CD28 or 4-1BB co-stimulatory domains, and a CD3ζ intracellular domain, and were cloned into a pMIG retroviral vector. For bioluminescence imaging studies, T cells were co-transduced with MI-FLuc-IRES-mCherry, which was a gift from Xiaoping Sun (Addgene plasmid #75020; http://n2t.net/addgene:75020; RRID: Addgene_75020). All humanized CARs were similarly cloned as above using humanized domains, with the exception that CARs containing CD28 co-stimulatory domains were paired with a CD28 transmembrane domain. Humanized CARs were cloned into pLenti6.3 lentiviral vector (ThermoFisher) under the control of a cytomegalovirus promoter. For bioluminescence imaging studies, T cells were co-transduced with pCDH-EF1-Luc2-P2A-tdTomato, which was a gift from Kazuhiro Oka (Addgene plasmid #72486; http://n2t.net/addgene:72486; RRID: Addgene_72486). Expression of all CARs was determined by flow cytometry staining for the Myc-tag using an anti-Myc tag antibody (9B11, PE conjugate, Cell Signaling) with analysis on a BD LSR-II flow cytometer.

### Retrovirus production and engineering of murine CAR T cells

To produce ecotropic retrovirus for murine T cell transduction, Phoenix-ECO cells were transfected with a 1:3 ratio of pCL-Eco and transfer plasmid using a CalPhos Mammalian Transfection Kit (Takara Bio). Primary murine T cells were isolated from spleens of WT or CD45.1^+^ C57BL/6 mice using the EasySep Mouse CD8^+^ T Cell Isolation Kit or EasySep Mouse T Cell Isolation Kit (StemCell Technologies) and activated for 48 h on six-well non-tissue culture (TC)-treated plates precoated with 0.5 mg ml^−1^ anti-CD3 (BioXCell, Clone 2C11) and 5 mg ml^−1^ anti-CD28 (BioXCell, Clone 37.51) at 10^6^ cells ml^−1^ with 5 ml per well. T cells were cultured in complete RPMI with 1 mM sodium pyruvate, 0.05 mM β-mercaptoethanol and 1× minimal essential medium non-essential amino acids (ThermoFisher) supplemented with 10 ng ml^−1^ murine IL-2 (BioLegend). Following activation, T cells were transduced by spinfection at 3 × 10^6^ cells per well in complete T cell medium for 90 min at 1,100*g* and 32 °C with 10 ng ml^−1^ polybrene (Sigma) on non-TC-treated plates pre-coated with 15 mg ml^−1^ RetroNectin (Takara Bio). CAR expression was assayed via flow cytometry 24 h post-transduction; T cells were maintained at a cell density of 10^6^ cells ml^−1^, then used for adoptive transfer or in vitro assays at 48 h post-transduction.

### Lentivirus production and engineering of human CAR T cells

For humanized CARs, vesicular stomatitis virus G-pseudotyped lentivirus was produced via transfection of 293T cells with psPAX2, pMD2.G and transfer plasmid in a 2:1:2 ratio using Effectene Transfection Reagent (QIAGEN). Primary human T cells were isolated from buffy coats from healthy donors (Massachusetts General Hospital Blood Donor Center) using the EasySep Human CD8^+^ T Cell Isolation Kit or EasySep Human T Cell Isolation Kit (StemCell Technologies) and activated for 48 h using Dynabeads Human T-Activator CD3/CD28 (ThermoFisher) at a 3:1 bead-to-cell ratio. Human T cells were cultured in complete RPMI supplemented with 30 U ml^−1^ human IL-2 (PeproTech). Following activation, T cells were transduced and stained as described above, and used for adoptive transfer or in vitro studies at 48–72 h post-transduction.

### Amph-FITC labelling and cytotoxicity studies

For in vitro labelling of tumour cells with amph-FITC, tumour cells were washed with 1× phosphate-buffered saline (PBS) and incubated at a cell density of 10^6^ cells ml^−1^ for 30 min at 37 °C with DSPE-PEG_*x*_-FITC, where *x* = 0, 1, 2, 3.4 or 10 kDa (Creative PEGworks) in PBS. For co-cultures, target cells were labelled simultaneously with 100 nM amph-FITC and CellTrace Violet (ThermoFisher); 20,000 target cells were seeded per well in a 96-well flat-bottom plate with CAR T cells at the indicated E:T ratios. Following a 16 h incubation, cells were stained with SYTOX red (ThermoFisher) for flow cytometry, and IFN-γ in the supernatant was quantified using a Mouse IFN gamma Uncoated ELISA Kit (Invitrogen).

### Biodistribution analysis

To assess biodistribution of amph-FITC following i.t. injection, C57BL/6 mice (6–8 weeks, Jackson Laboratory, *n* = 5) were inoculated s.c. with 10^6^ B16F10 tumour cells. Once tumours were ~25 mm^2^ in area, 10 nmol DSPE-PEG_2k_-FITC (Creative PEGworks) was injected intratumourally. After 24 h, tissues (tumour, liver, kidney, spleen, bone marrow and both axillary and inguinal LNs) were homogenized in ethanol (pH 8.0–8.5) and supernatant fluorescence was quantified on a FlexStation 3 plate reader (Molecular Devices, excitation 495 nm, emission 519 nm).

For cellular localization studies, MC38 tumour-bearing mice were injected intratumourally with 10 nmol DSPE-PEG_2k_-FITC, and 24 h later tumours were enzymatically digested at 37 °C for 30 min with 1 mg ml^−1^ collagenase D and 0.2 mg ml^−1^ DNase I in complete RPMI medium before mechanical dissociation through a 70 µm cell strainer. Samples were stained using the following antibodies: PE-Cy7 anti-CD45 (clone 30-F11, BioLegend), BV605 anti-CD11b (clone M1/70, BioLegend), PE anti-CD11c (clone N418, BioLegend), BV711 anti-mouse CD3 (clone 17A2, BioLegend), BV421 anti-CD8α (clone 53-6.7, BioLegend) and Alexa Fluor 647 anti-FITC (Jackson ImmunoResearch) and were analysed on a BD LSRFortessa flow cytometer.

### In vivo therapy studies

All animal work was conducted under an MIT Division of Comparative Medicine institute animal care, used a committee-approved animal protocol by the Committee of Animal Care at MIT, and used a committee-approved protocol in accordance with federal, state and local guidelines.

C57BL/6 WT, CD45.1^+^, Batf3 knockout (KO) and Rag1 KO mice (Jackson Laboratory, 6–8 weeks old) were inoculated with 10^6^ B16F10, 10^6^ MC38 or 3 × 10^6^ CT-2A tumour cells s.c. at the flank. Once tumours grew to ~25 mm^2^ in area, mice underwent a non-myeloablative 5 Gy dose of TBI administered by a [^137^Cs] gamma radiation source, 24 h before adoptive transfer of 10^7^ CD45.1^+^ CAR T cells intravenously (i.v.) via the tail vein. One day later, mice were vaccinated s.c. at the tail base with 10 nmol DSPE-PEG_2k_-FITC (Creative PEGworks) and 25 µg cyclic di-GMP (Invivogen), and vaccinated for three total doses, once weekly. Injection (i.t.) of 10 nmol amph-FITC was administered beginning the day after the first vaccination, and continuing every 3 days.

For xenograft studies, 6- to 12-week-old NOD.Cg-*Prkdc*^*scid*^*IL2rg*^*tm1Wjl*^/SzJ (NSG) mice (Jackson Laboratory) were inoculated with 3 × 10^6^ MSTO-211H cells s.c. at the flank. Once tumours grew to ~25 mm^2^, mice were adoptively transferred with 10^7^ CAR T cells i.v. via the tail vein. Injection (i.t.) of 10 nmol amph-FITC was administered beginning the day after adoptive transfer, and continuing every 3 days.

For all studies, tumour progression was monitored by caliper measurements. Survival was evaluated over time until tumours exceeded 200 mm^2^ in area, were severely ulcerated >1 week or experienced >20% weight loss. To monitor trafficking of FLuc-expressing CAR T cells, mice were injected s.c. at the scruff with 150 mg kg^−1^ d-luciferin K^+^ salt (Perkin-Elmer) and imaged on a Xenogen IVIS Spectrum. Before imaging, C57BL/6 mice were depilated on their back to improve signal sensitivity.

### Flow cytometry

For quantification of murine CAR T and endogenous T cells in the peripheral blood, tumour and spleen, tissues were homogenized through a 70 µm cell strainer (with the exception of MC38 tumours, which required enzymatic digestion as described above). Blood and spleen samples were incubated with ACK lysing buffer (Gibco) to isolate peripheral blood mononuclear cells and splenocytes, respectively. Samples were then stained with PE anti-CD45.1 (clone A20, BioLegend), BUV805 anti-CD8α (clone 53-6.7, BioLegend), Alexa Fluor 647 anti-CD4 (clone GK1.5, BioLegend), APC-Cy7 anti-CD45.2 (clone 104, BioLegend) and LIVE/DEAD Fixable Aqua (ThermoFisher) and analysed on a BD LSRFortessa flow cytometer.

For antigen uptake studies, tumours and LNs were stained with Zombie Aqua Fixable Viability Kit (BioLegend), BUV395 anti-CD45 (clone 30-F11, BD Biosciences), BV421 anti-CD103 (clone 2E7, BioLegend), BV605 anti-Ly6C (clone HK1.4, BioLegend), BV711 anti-F4/80 (clone BMB, BioLegend), BV785 anti-CD11b (clone M1/70, BioLegend), Alexa Fluor 700 anti-CD86 (clone GL1, BioLegend), PE-Cy7 anti-I-A/I-E (clone M5/114.15.2, BioLegend), APC anti-CD24 (clone 30-F1, BioLegend), APC-Cy7 anti-CD11c (clone N418, BioLegend), PE-Cy5 anti-CCR7 (clone 4B12, BioLegend), PerCP-Cy5.5 anti-CD169 (clone 3D6.112, BioLegend) and BUV737 anti-CD8α (clone 53-6.7, BD Biosciences).

For quantification of human CAR T cells in the peripheral blood, spleen and tumour, tissues were processed as described above, then stained with LIVE/DEAD Aqua, BUV496 anti-CD4 (clone SK3/Leu3a, BD Biosciences), BUV805 anti-CD8α (clone SK1, BD Biosciences), PE anti-myc tag (clone 9B11, Cell Signaling) and APC-Cy7 anti-CD45 (clone 2D1, BioLegend).

### ELISPOT

Spleens were processed as described above. CT-2A and B16F10 target cells were pre-treated with 100 U ml^−1^ and 500 U ml^−1^ murine IFN-γ (Abcam), respectively for 16 h, then irradiated at 120 Gy with a [^137^Cs] gamma radiation source. Splenocytes (several dilutions starting with 10^6^ cells per well) were co-cultured with 25,000 target cells per well for 16 h, and ELISPOT was performed using a Mouse IFN-γ ELISPOT Set (BD Biosciences) according to the manufacturer’s instructions.

### Immunohistochemistry and immunofluorescence

Tissues (tumour, lungs, liver and kidney) were fixed in 4% paraformaldehyde in PBS for 24 h at 4 °C. For immunofluorescence studies, tumours were embedded in 3% low gelling temperature agarose (Sigma-Aldrich) before processing into 100 µm sections on a Leica VT1000S vibratome. Tissue sections were permeabilized with 2% bovine serum albumin and 0.2% Triton-X 100 in PBS for 1 h at 37 °C before staining with Alexa Fluor 647- or DyLight 405-conjugated anti-FITC (Jackson ImmunoResearch) for 16 h at 37 °C, followed by several PBS washes. Confocal microscopy was performed on an Olympus FV1200 Confocal Laser Scanning Microscope. For amph-FITC tumour distribution immunohistochemistry, paraformaldehyde-fixed samples were paraffin-embedded and 10 µm sections were obtained. Sections were stained with anti-myc tag (9B11, Cell Signaling) or anti-CD45.1 (A20, BioLegend). For immunohistochemistry studies, tumours were frozen in optimal cutting temperature compound and processed into 14-μm-thick sections on a Leica CM1950 cryostat. Tissue sections were permeabilized with 2% bovine serum albumin and 0.2% Triton-X 100 in PBS for 2 h at room temperature before staining with biotinylated anti-Trp1 (TA99) (gift from Dr Dane Wittrup at MIT), BV421-conjugated streptavidin (Biolegend), Alexa Fluor 647-conjugated anti-FITC (clone SPM395, Novus Biologicals) and CellMask Orange Plasma Membrane Stain (Invitrogen). Imaging was performed on a Leica SP8 Spectral Confocal Microscope.

### Statistical analysis

Statistical analyses were performed using GraphPad Prism 9. All data are presented as mean ± standard deviation (s.d.). To assess statistical significance, the following tests were used: between two groups, unpaired two-tailed Student’s *t*-test or Mann–Whitney *U* test; for multiple comparisons, two-way analysis of variance (ANOVA) with Tukey’s multiple comparisons; for repeated measures over a time course, two-way ANOVA with repeated measures; for Kaplan–Meier survival curves, log-rank (Mantel–Cox) test. All *P* values are provided in the figures or in their legends.

### Reporting summary

Further information on research design is available in the Nature Portfolio Reporting Summary linked to this article.

## Supplementary information

Supplementary Information Supplementary Figs. 1–5.

Reporting Summary

## Data Availability

The main data supporting the results in this study are available within the paper and its Supplementary Information. Any data supporting the findings of this study are also available from the corresponding authors on reasonable request. Source data are provided with this paper.

## Source data

Source Data for All Figures Source data for all figures.
