# Supplementary material for: Universal redirection of CAR T cells against solid tumours via membrane-inserted ligands for the CAR
Source: Nat Biomed Eng. 2023 Jun 8;7(9):1113–28. doi: 10.1038/s41551-023-01048-8 (PMC10504084; doi:10.1038/s41551-023-01048-8)
Supplement: Supplementary file 1 — Supplementary Figs. 1–5. [file 41551_2023_1048_MOESM1_ESM.pdf]

nature biomedical engineering

Article https://doi.org/10.1038/s41551-023-01048-8

Universal redirection of CAR T cells against solid tumours via membrane-inserted ligands for the CAR

In the format provided by the authors and unedited

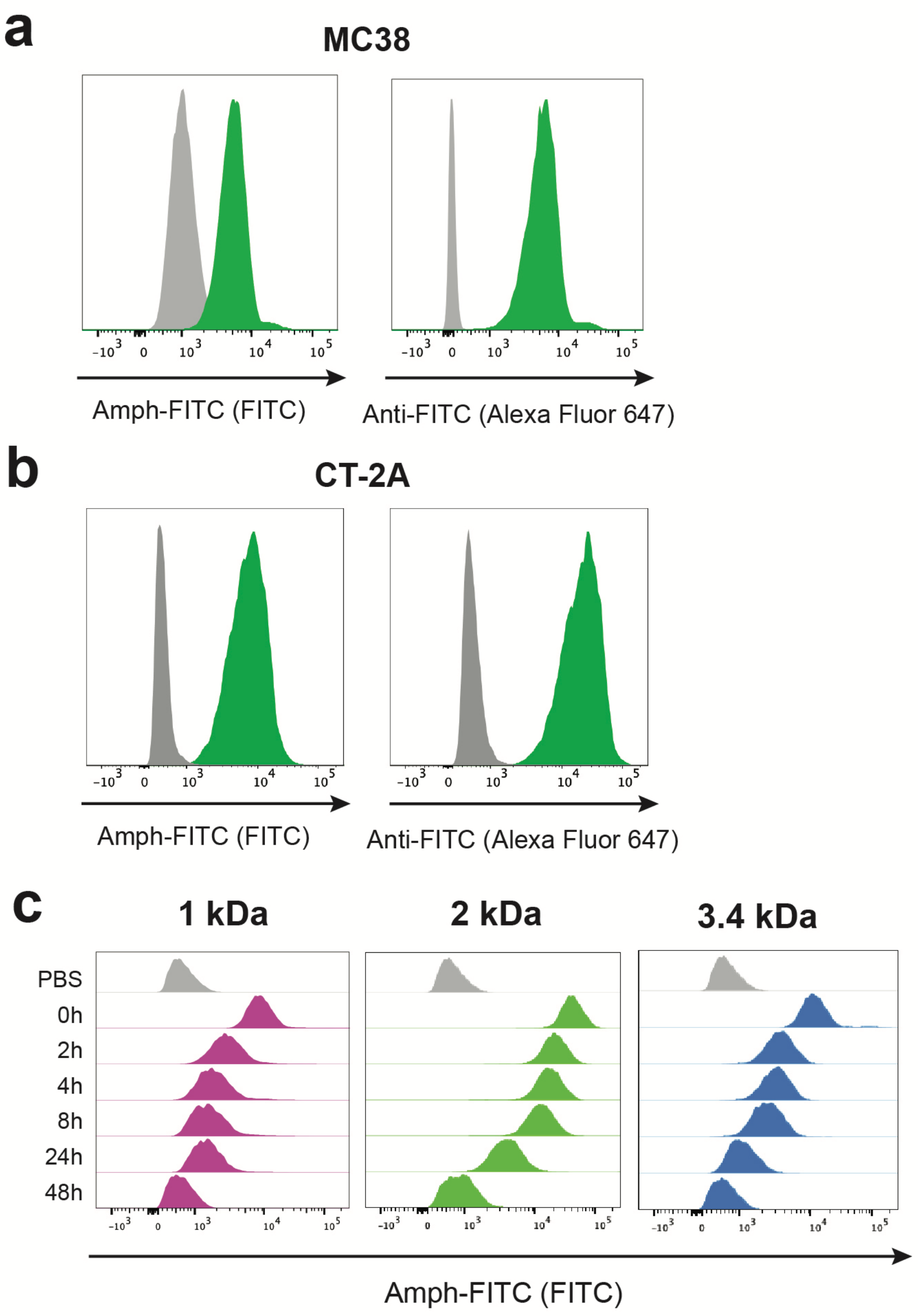

Supplementary Fig. 1 | Characterization of kinetics of amph-FITC tagging. a,b MC38 murine colon carcinoma cells (**a**) and CT-2A murine glioma cells (**b**) were incubated with amph-FITC at 100 nM for 30 min at 37°C in PBS, stained with an anti-FITC antibody, and analyzed by flow cytometry. Shown are representative histograms of total FITC (left) and anti-FITC signals (right). **c** Kinetic analysis of amph-FITC MFI of amph-FITC+ B16F10 cultured in RPMI following tagging.

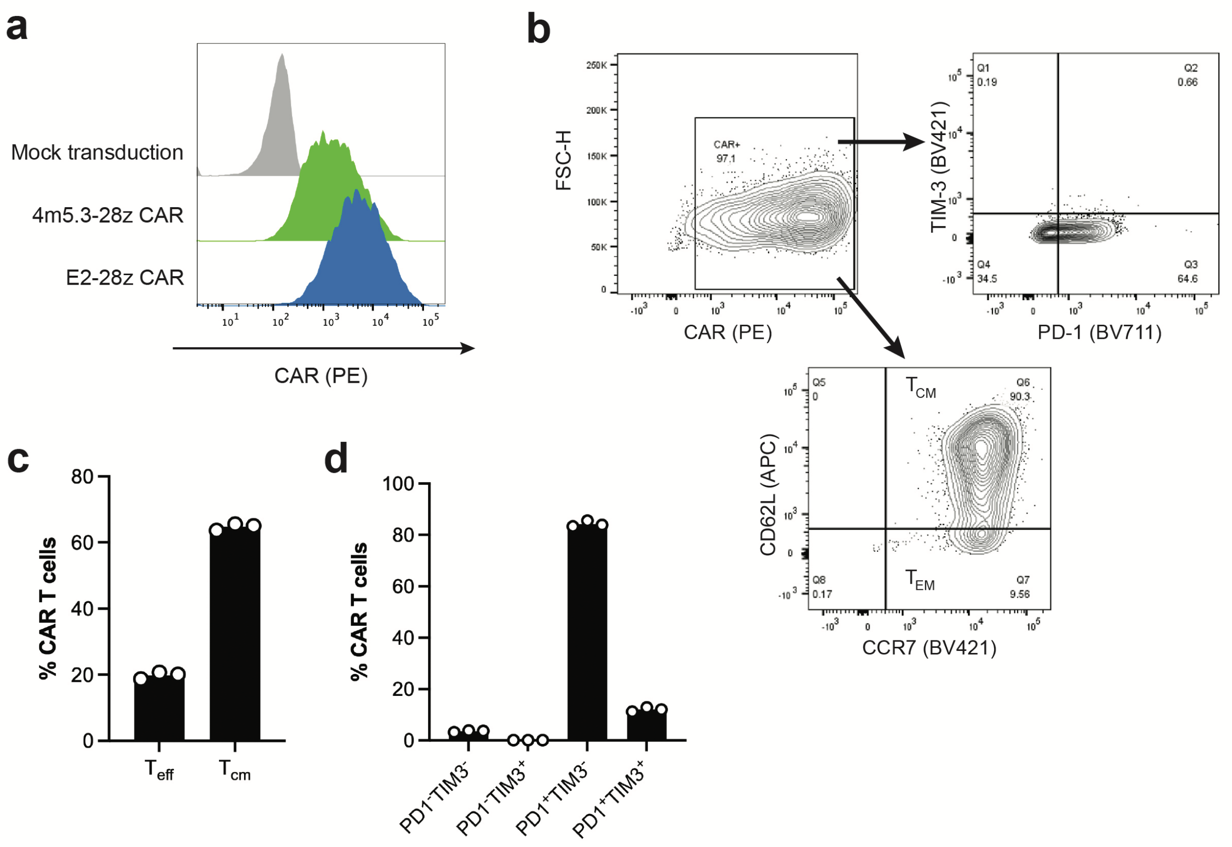

Supplementary Fig. 2 | Murine T cells are effectively transduced to express FITC-specific CARs. a CAR expression in murine CD8+ 4m5.3-28z and E2-28z FITC CAR T cells, detected by staining of Myc tag. **b** Gating for exhaustion and memory phenotypes of FITC CAR T cells two days post-transduction. **c** CAR T cell phenotypes prior to adoptive transfer. (*n* = 3 technical replicates) **d** Mean PD-1/TIM-3 expression by CAR T cells prior to adoptive transfer. (*n* = 3 technical replicates)

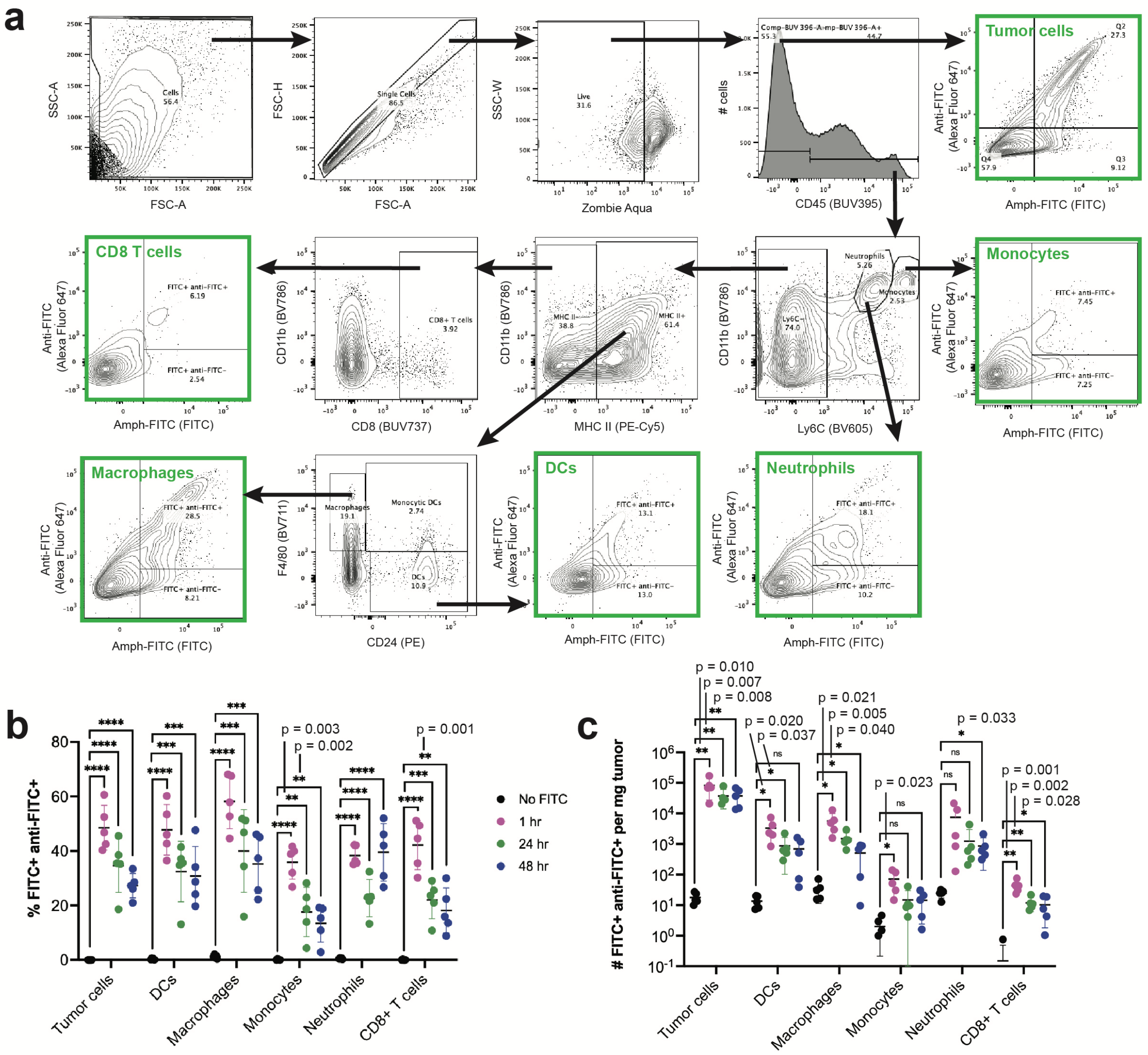

Supplementary Fig. 3 | Biodistribution of intratumorally injected amph-FITC. **a** Gating strategy for tumor immunophenotyping. **b**,**c** Percent FITC⁺ anti-FITC⁺ double-positive cells (**b**) and density of FITC⁺ anti-FITC⁺ cells (**c**) in B16F10 tumors in C57BL/6 mice lymphodepleted with 5 Gy TBI at 1, 24, and 48 hr following amph-FITC injection (n = 5 animals/group). p values were determined by unpaired Student's t test. Error bars represent standard deviation. *$p < 0.05$, **$p < 0.01$, ***$p < 0.001$, ****$p < 0.0001$.

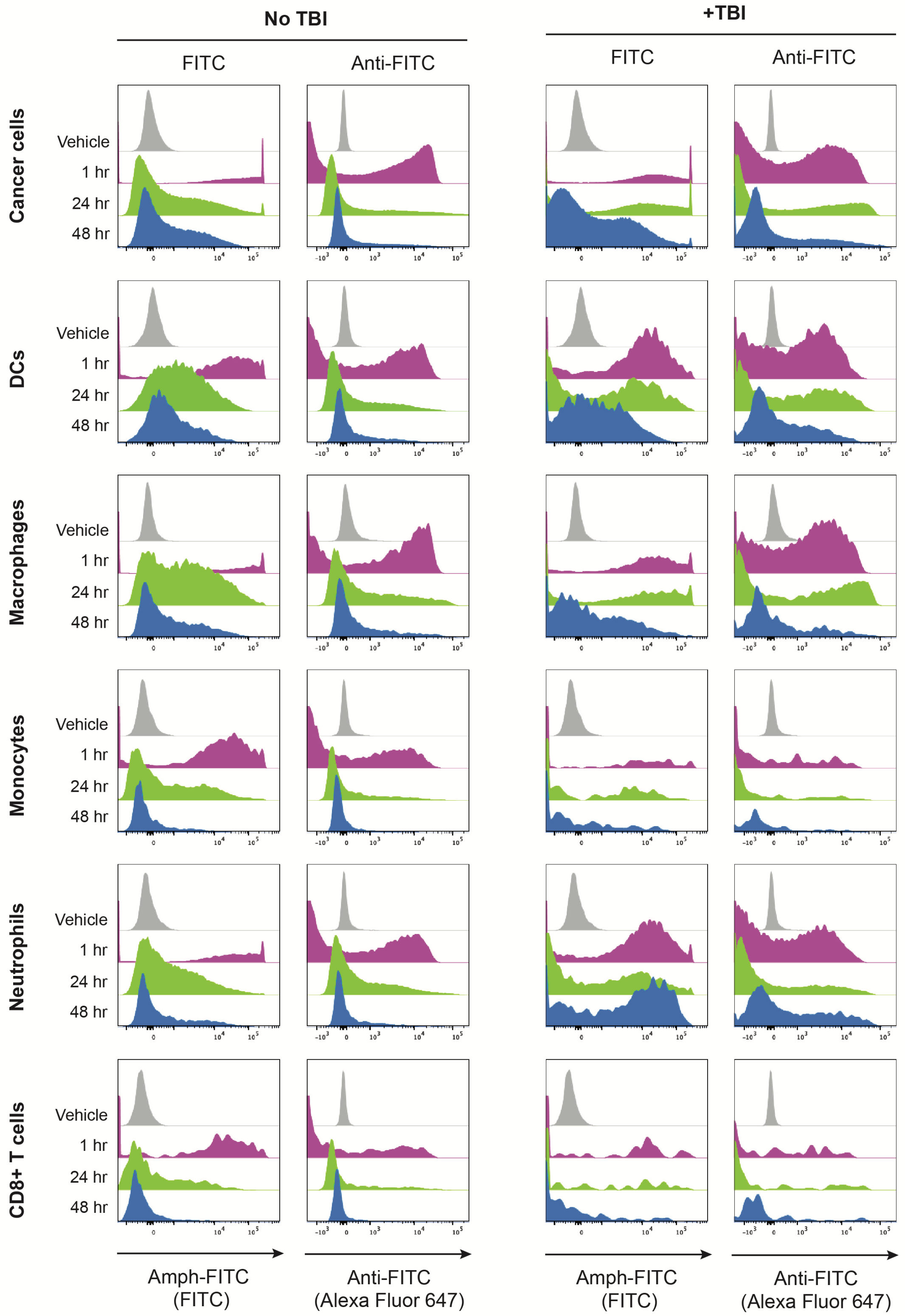

Supplementary Fig. 4 | Sample histograms of labeled cell types. Representative histograms of FITC and anti-FITC signal of cancer cells and immune cell subsets in lymphoreplete (left) and lymphodepleted (TBI, right) mice at 1, 24, and 48 hr following amph-FITC injection.

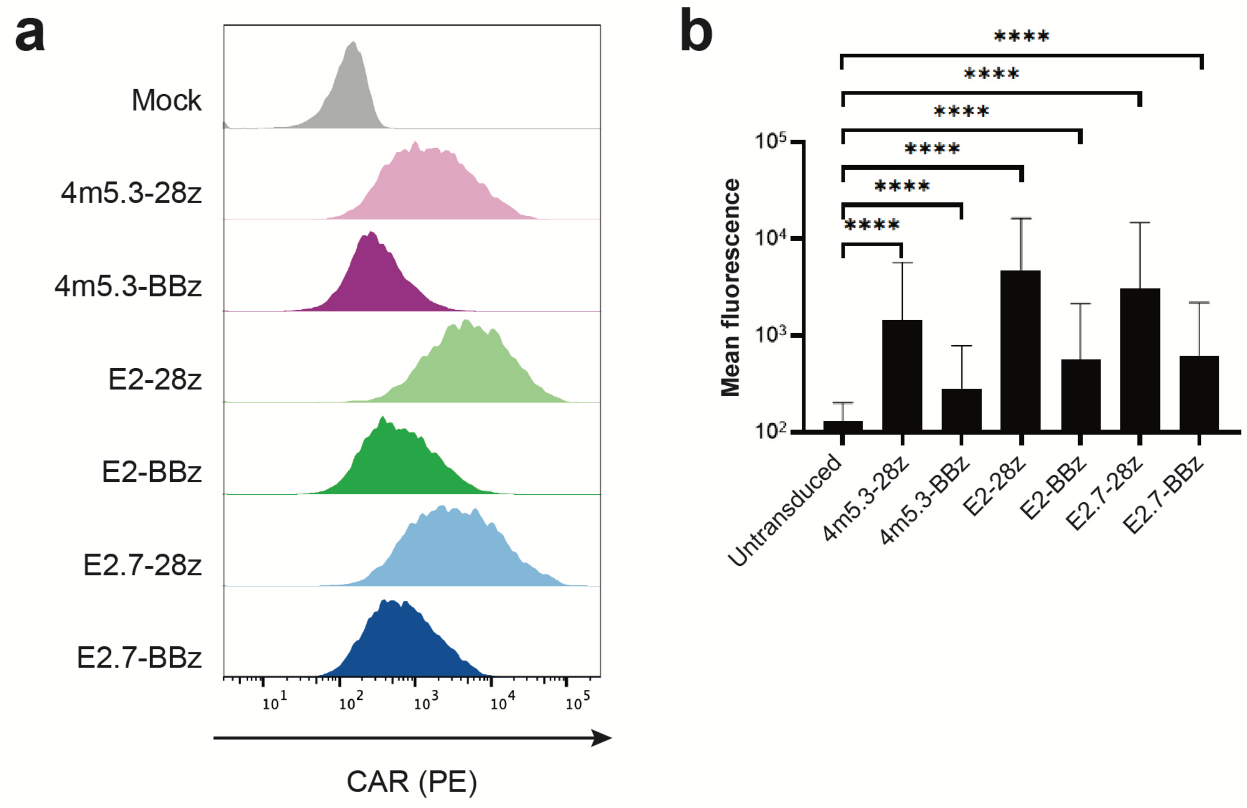

Supplementary Fig. 5 | Expression of CAR variants in murine CD8+ T cells. a, b Histograms (**a**) and quantification (mean ± SD) (**b**) of CAR expression 24 hr post-transduction of primary murine CD8+ T cells for FITC scFvs of varying affinity and with CD28 vs. 4-1BB costimulatory domains. *P* values were determined by unpaired Student's *t* test. Error bars represent standard deviation. ****$p < 0.0001$.
