# Supplementary material for: Universal redirection of CAR T cells against solid tumours via membrane-inserted ligands for the CAR
Source: Nat Biomed Eng. 2023 Jun 8;7(9):1113–28. doi: 10.1038/s41551-023-01048-8 (PMC10504084; doi:10.1038/s41551-023-01048-8)
Supplement: Supplementary file 2 — Reporting Summary [file 41551_2023_1048_MOESM2_ESM.pdf]

nature portfolio

Corresponding author(s): Darrell J. Irvine

Last updated by author(s): Jan 5, 2023

Reporting Summary

Nature Portfolio wishes to improve the reproducibility of the work that we publish. This form provides structure for consistency and transparency in reporting. For further information on Nature Portfolio policies, see our Editorial Policies and the Editorial Policy Checklist.

Statistics

For all statistical analyses, confirm that the following items are present in the figure legend, table legend, main text, or Methods section.

n/a	Confirmed	
☐	☒	The exact sample size (*n*) for each experimental group/condition, given as a discrete number and unit of measurement
☐	☒	A statement on whether measurements were taken from distinct samples or whether the same sample was measured repeatedly
☐	☒	The statistical test(s) used AND whether they are one- or two-sided<br>*Only common tests should be described solely by name; describe more complex techniques in the Methods section.*
☐	☒	A description of all covariates tested
☐	☒	A description of any assumptions or corrections, such as tests of normality and adjustment for multiple comparisons
☐	☒	A full description of the statistical parameters including central tendency (e.g. means) or other basic estimates (e.g. regression coefficient) AND variation (e.g. standard deviation) or associated estimates of uncertainty (e.g. confidence intervals)
☐	☒	For null hypothesis testing, the test statistic (e.g. F, t, r) with confidence intervals, effect sizes, degrees of freedom and P value noted<br>*Give P values as exact values whenever suitable.*
☒	☐	For Bayesian analysis, information on the choice of priors and Markov chain Monte Carlo settings
☒	☐	For hierarchical and complex designs, identification of the appropriate level for tests and full reporting of outcomes
☒	☐	Estimates of effect sizes (e.g. Cohen's *d*, Pearson's *r*), indicating how they were calculated

Our web collection on statistics for biologists contains articles on many of the points above.

Software and code

Policy information about availability of computer code

Data collection	FACS data were obtained using the BD FACSDiva software. ELISA plates and plate-based fluorescent/absorbance experiments were measured using the Molecular Devices Flexstation 3 instrument and software. ELISPOT measurements were taken using the CTL Immunospot Analyzer and associated software. Confocal images were taken on a Olympus FV1200 confocal microscope and associated software. IVIS data were collected on the IVIS Spectrum In Vivo Imaging system.
Data analysis	FlowJo 10.8.1 was used for the analysis of FACS data. GraphPad Prism 9.2.0 was used for plotting and statistical analysis. Fiji (Image J v2.1.0) was used for histology-image analysis. Excel, Word and Powerpoint from Microsoft Office (version 16.49) were used to draft the manuscript. IVIS data were analysed using Living Image v4.5.

For manuscripts utilizing custom algorithms or software that are central to the research but not yet described in published literature, software must be made available to editors and reviewers. We strongly encourage code deposition in a community repository (e.g. GitHub). See the Nature Portfolio guidelines for submitting code & software for further information.

Data

Policy information about availability of data

All manuscripts must include a data availability statement. This statement should provide the following information, where applicable:

- Accession codes, unique identifiers, or web links for publicly available datasets
- A description of any restrictions on data availability
- For clinical datasets or third party data, please ensure that the statement adheres to our policy

The main data supporting the results in this study are available within the paper and its Supplementary Information. Any data supporting the findings of this study are also available from the corresponding authors on reasonable request. Source data are provided with this paper.

Human research participants

Policy information about studies involving human research participants and Sex and Gender in Research.

Reporting on sex and gender	The study did not involve human research participants.
Population characteristics	—
Recruitment	—
Ethics oversight	—

Note that full information on the approval of the study protocol must also be provided in the manuscript.

Field-specific reporting

Please select the one below that is the best fit for your research. If you are not sure, read the appropriate sections before making your selection.

☒ Life sciences ☐ Behavioural & social sciences ☐ Ecological, evolutionary & environmental sciences

For a reference copy of the document with all sections, see nature.com/documents/nr-reporting-summary-flat.pdf

Life sciences study design

All studies must disclose on these points even when the disclosure is negative.

Sample size	Sample sizes were selected to enable a 20% difference between groups to be detected with a power of 80%, on the basis of preliminary studies and of prior work from our laboratory in similar experiments.
Data exclusions	No data were excluded.
Replication	The xperiments were repeated to confirm reproducibility, as indicated in the figure captions.
Randomization	For tumour studies, the animals were randomized to groups to achieve equivalent mean tumor sizes at the start of the treatment.
Blinding	No blinding was done in the study, owing to differences in treatment administration between conditions.

Reporting for specific materials, systems and methods

We require information from authors about some types of materials, experimental systems and methods used in many studies. Here, indicate whether each material, system or method listed is relevant to your study. If you are not sure if a list item applies to your research, read the appropriate section before selecting a response.

Materials & experimental systems

n/a	Involved in the study	
☐	☒	Antibodies
☐	☒	Eukaryotic cell lines
☒	☐	Palaeontology and archaeology
☐	☒	Animals and other organisms
☒	☐	Clinical data
☒	☐	Dual use research of concern

Methods

n/a	Involved in the study	
☒	☐	ChIP-seq
☐	☒	Flow cytometry
☒	☐	MRI-based neuroimaging

Antibodies

Antibodies used

In vitro T cell activation: anti-mCD3 (BioXCell, Cat. #BE0001-1, Clone 145-2C11); anti-mCD28 (BioXCell, Cat. #BE0015-1, Clone 37.51). Antibodies were used at concentrations as described in the Methods.

Flow cytometry: anti-Myc tag (Cell Signaling, Cat. #3739, Clone 9B11); anti-mCD45 (Biolegend, Cat. #103114 , Clone 30-F11); anti-CD11b (Biolegend, Cat. #101237, Clone M1/70); anti-CD11c (Biolegend, Cat. #117308, Clone N418); anti-mCD3 (Biolegend, Cat. #100241, Clone 17A2); anti-mCD8a (Biolegend, Cat. #100737, Clone 53-6.7); anti-FITC (Jackson ImmunoResearch, Cat. #200-602-037, 1:500 dilution); anti-mCD45.1 (Biolegend, Cat. #110708, Clone A20); anti-mCD8a (Biolegend, Cat. 100712, Clone 53-6.7); anti-mCD4 (Biolegend, Cat. #100424, Clone GK1.5); anti-mCD45.2 (Biolegend, Cat. #109824, Clone 104); anti-mCD45 (BD Biosciences, Cat. #564279, Clone 30-F11); anti-mCD103 (Biolegend, Cat. #121421, Clone 2E7); anti-Ly-6C (Biolegend, Cat. #128035, Clone HK1.4); anti-mF4/80 (Biolegend, Clone BMB); anti-CD11b (Biolegend, Cat. #101243, Clone M1/70); anti-mCD86 (Biolegend, Cat. #105024, Clone GL-1); anti-mMHC-II (Biolegend, Cat. #107630, Clone M5/114.15.2); anti-mCD24 (Biolegend, Cat. #138506, Clone 30-F1); anti-mCD11c (Biolegend, Cat. #117324, Clone N418); anti-hCD4 (Biolegend, Cat. #120114, Clone 4B12); anti-mCD169 (Biolegend, Cat. #142410, Clone 3D6.112); anti-mCD8a (BD Biosciences, Cat. #612759, Clone 53-6.7); anti-hCD4 (BD Biosciences, Cat. #612936, Clone SK3); anti-hCD8a (BD Biosciences, Cat. #612889, Clone SK1); anti-hCD45 (Biolegend, Cat #368516, Clone 2D1). All antibodies were used at a 1:200 dilution unless indicated above.

Histology: anti-FITC (Jackson ImmunoResearch, Cat. #200-472-037, 1:400 dilution); streptavidin (Biolegend, Cat. #405226, 1:100 dilution); anti-FITC (Novus Biologicals, Cat. #NBP3-08492AF647, Clone SPM395, 1:400 dilution).

All other antibodies used for ELISA/ELISPOT or cell isolation purposes were from commercial kits cited in Methods.

Validation

All antibodies other than TA99 were validated by the vendors: Biolegend, BD Biosciences, ThermoFisher, Abcam and BioXCell. TA99 was synthesized in-house and validated by a flow-cytometry B16F10 binding assay and used in a previous publication (Momin et al., Sci. Transl. Med. 11, eaaw2614 (2019)).

Eukaryotic cell lines

Policy information about cell lines and Sex and Gender in Research

Cell line source(s)

CT-2A cells were a gift from Dr. Thomas Seyfried, Boston College. MC38 cells were a gift from Dr. K. Dane Wittrup, MIT. Phoenix-ECO, B16F10 and MSTO-211H cells were purchased from ATCC.

Authentication

Each cell line was maintained separately and stocked in early passages to minimize contamination and to preserve cell identity.

Mycoplasma contamination

The cell lines were periodically tested and confirmed to be free of mycoplasma contamination.

Commonly misidentified lines (See ICLAC register)

No commonly misidentified cell lines were used.

Animals and other research organisms

Policy information about studies involving animals; ARRIVE guidelines recommended for reporting animal research, and Sex and Gender in Research

Laboratory animals

C57BL/6, Batf3–/–, RAG1–/–, CD45.1 C57BL/6 and NOD.Cg-PrkdcscidIL2rgtm1Wjl/SzJ (NSG) mice were all obtained from Jackson Laboratories. Mice were between 6 and 12 weeks old at the start of all studies and weighed approximately 24–28 g (male mice) or 17–19 g (female mice).

Wild animals

The study did not involve wild animals.

Reporting on sex

All studies using C57BL/6 mice employed female mice. Studies with NSG mice used a combination of male and female mice, evenly distributed across conditions.

Field-collected samples

The study did not involve samples collected from the field.

Ethics oversight

All animal studies and procedures were carried out following federal, state and local guidelines under an institutional-animal-care-

Ethics oversight | and-use-committee-approved animal protocol by the Committee of Animal Care at MIT.

Note that full information on the approval of the study protocol must also be provided in the manuscript.

Flow Cytometry

Plots

Confirm that:

☒ The axis labels state the marker and fluorochrome used (e.g. CD4-FITC).

☒ The axis scales are clearly visible. Include numbers along axes only for bottom left plot of group (a 'group' is an analysis of identical markers).

☒ All plots are contour plots with outliers or pseudocolor plots.

☒ A numerical value for number of cells or percentage (with statistics) is provided.

Methodology

Sample preparation: Tumours and lymph nodes were mechanically processed through 70-µm nylon cell strainers to prepare single-cell suspensions. Peripheral blood was collected by submandibular bleeding into K2-EDTA tubes (Greiner-Bio) and red blood cells were lysed in ACK Lysis Buffer (Gibco). All samples were then resuspended in ice-cold PBS containing 1% (w/v) BSA and 2 mM EDTA (FACS buffer) with precision count beads (Biolegend, normalized to the weight of tissue per sample) before staining.

Instrument: Cells were analysed using BD FACS LSR Fortessa or BD FACS Symphony A3 flow cytometers.

Software: BD FACSDiva (BD Biosciences) was used for the collection of FACS data and FlowJo was used for data analysis. The collected data were plotted with statistical analysis by GraphPad Prism.

Cell population abundance: No sorting was used.

Gating strategy: Example gating strategies are provided in the Supplementary information.

☒ Tick this box to confirm that a figure exemplifying the gating strategy is provided in the Supplementary Information.
